# Magmatic immiscibility and the origin of magnetite-(apatite) iron deposits

**DOI:** 10.1038/s41467-023-43655-8

**Published:** 2023-12-19

**Authors:** Dorota K. Pietruszka, John M. Hanchar, Fernando Tornos, Richard Wirth, Nathan A. Graham, Kenneth P. Severin, Francisco Velasco, Matthew Steele-MacInnis, Wyatt M. Bain

**Affiliations:** 1 Department of Earth Sciences, Memorial University of Newfoundland, St. John’s, NL A1B 3X5 Canada; 2 Instituto de Geociencias (CSIC-UCM), Severo Ochoa 7, 28040 Madrid, Spain; 3 GFZ German Research Centre for Geosciences, Section 3.5 Interface Geochemistry, Telegrafenberg, Potsdam, 14473 Germany; 4 Department of Geosciences, University of Alaska Fairbanks, Fairbanks, AK 99775 USA; 5 Departamento de Mineralogía y Petrología, Universidad del País Vasco UPV/EHU, 48080 Bilbao, Spain; 6 Department of Earth & Atmospheric Sciences, University of Alberta, Edmonton, AB T6G 2E3 Canada; 7 British Columbia Geological Survey, Ministry of Energy, Mines, and Low Carbon Innovation, Victoria, BC V8T 4J1 Canada

**Keywords:** Economic geology, Petrology

## Abstract

The origin of magnetite-(apatite) iron deposits (MtAp) is one of the most contentious issues in ore geology with competing models that range from hydrothermal to magmatic processes. Here we report melt inclusions trapped in plagioclase phenocrysts in andesite hosting the MtAp mineralization at El Laco, Chile. The results of our study reveal that individual melt inclusions preserve evidence of complex processes involved in melt immiscibility, including separation of Si- and Fe-rich melts, the latter hosting Cu sulfide-rich, phosphate-rich, and residual C-O-HFSE-rich melts, with their melting temperature at 1145 °C. This association is consistent with the assemblages observed in the ore, and provides a link between silicate and Fe-P-rich melts that subsequently produced the magnetite-rich magmas that extruded on the flanks of the volcano. These results strongly suggest that the El Laco mineralization was derived from crystallization of Fe-P-rich melts, thus providing insight into the formation of similar deposits elsewhere.

## Abstract (web summary)

Magnetite-apatite (MtAp) deposits are an important source of iron and critical metals, yet their ore genesis is poorly understood. Here, authors propose that the separation of multiple melts during magma ascent leads to the formation of MtAp deposits.

## Introduction

**Fig. 1:**
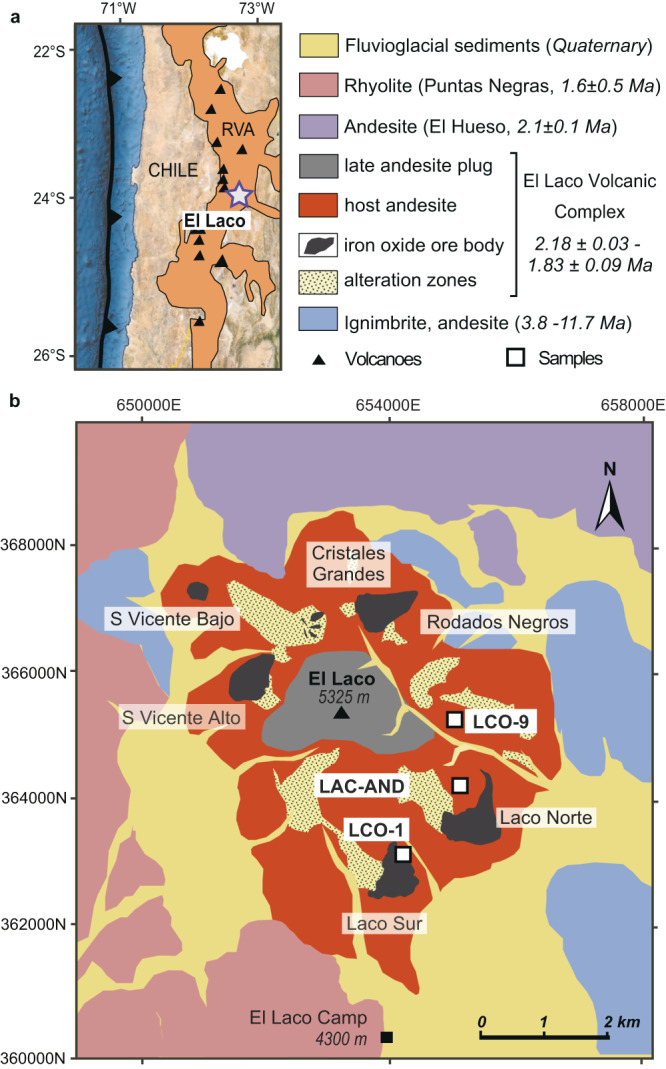
Location of the El Laco magnetite-(apatite) deposit. **a** Regional location of El Laco deposit, Chile. RVA—recent volcanic arc. **b** Geological map of El Laco deposit with sample locations. UTM zone and band 19 K. **a** and **b** modified from Tornos, F., Velasco, F. & Hanchar, J. M. Iron-rich melts, magmatic magnetite, and superheated hydrothermal systems: The El Laco deposit, Chile. Geology 44, 427–430 (2016). doi:10.1130/G37705.1^19^ in accordance with the GSA’s fair use policy.

The El Laco volcanic complex (ELVC) is located in the N-S trending Central Volcanic Zone (CVZ) in the Andean Cordillera of northern Chile (Fig. 1). The arc has been active from the Oligocene, achieving the peak of magmatic activity from the middle-Miocene to Pliocene^1^. At that time, tectonic thickening connected with compressional contraction of a weak lithosphere resulted in the formation of a significantly thick crust up to 70–80 km^1,2^. The crustal isotopic composition of Neogene-Quaternary volcanic rocks in the region favors significant contamination of arc magmas by different crustal rocks of Paleozoic and Mesozoic basement or by mingling with crustal felsic magmas^3–5^.

The ELVC is in the southern part of CVZ and its andesitic rocks are isotopically and geochemically similar to other adjacent volcanoes in CVZ^6,7^. The andesite of the ELVC shows strongly crustal radiogenic isotope signatures^3,5,7,8^ and has been dated (whole rock andesite Ar-Ar) at 2.18 ± 0.03 to 1.83 ± 0.09 Ma^9^. The ELVC hosts several bodies of magnetite-(apatite) (MtAp) mineralization with its geological resources exceeding 730 million metric tons at ~49 wt.% Fe^7^. The ore consists primarily of low-Ti magnetite and minor hematite, with scarce amounts of diopside, fluorapatite, and anhydrite—mostly forming stratabound sub-horizontal lenses of 1 to 10 m thick massive magnetite that encircle (~3 km in diameter) the volcanic edifice and underlying subvertical dikes, subvolcanic bodies and breccia pipes^7,10–12^ (Fig. 1b). The mineralization also includes friable, pyroclastic magnetite-hematite ore^10,13^ that hosts fragments of destinezite (Fe^3+^_2_(PO_4_)(SO_4_)(OH) · 6H_2_O) that have been interpreted as a crystallized immiscible melt^13–15^. The host andesite adjacent to the ore bodies is irregularly replaced by alkali-calcic alteration products comprising albite, diopside, magnetite, ilmenite, scapolite, fluorapatite, rutile, and locally minor pyrite^11,12^. Later acid-sulfate alteration that overprints the alkali-calcic alteration consists of predominantly alunite, kaolinite, gypsum, quartz, hematite, maghemite, and jarosite^7,16^.

El Laco is one of the youngest examples on Earth of MtAp mineralization with pristine and widespread outcrop exposure, minimal erosion, and an excellent drill core record. As such, this single locality has been at the focus of one of the most vigorous debates in the history of ore deposits. The genesis of this massive magnetite mineralization that resembles lava flows and related feeder dykes remains unclear despite numerous studies over six decades (e.g^10,13,16–20^.). Even today, there is no consensus on how MtAp deposits form, and models including magmatic, hydrothermal processes, or a combination of both end members have been proposed^17,19,21–25^. It is presently also unclear whether they are an independent style of mineralization, or genetically related to other systems, like iron oxide-copper-gold deposits (IOCG)^21,26^. Yet, these Paleoproterozoic to Holocene deposits are a significant source of iron in Chile, China, Iran, Peru, and Sweden, and have a significant resource potential for fluorite, phosphorus, REE, cobalt, and other critical metals^21,26^.

The unaltered host andesite of the ELVC is comprised of plagioclase, augite, and enstatite-pigeonite phenocrysts with accessory Fe-rich hornblende, biotite, apatite, Ti-rich magnetite, and glassy groundmass of rhyolitic composition. Plagioclase and clinopyroxene phenocrysts in the ELVC andesite host numerous melt inclusions that have textures with contrasting compositions indicating immiscibility between Fe-rich and Si-rich melts. These melt inclusions have previously been investigated^6,14^, but never at high-resolution and in as much detail as the present study. This and other widespread geologic and geochemical evidence at El Laco, led some previous researchers to propose that the magnetite mineralization was the product of the crystallization of an unusual Fe-rich melt^11,13,17–19,27^ that was formed through crustal contamination^7,8,28^ or direct melting of Fe-rich sedimentary units (e.g.^13,29^,) or evaporites^27^, and was later followed by exsolution of hydrothermal fluids leading to pervasive metasomatic alteration of the host rocks^6,7,19^. Other researchers have proposed alternative genetic hypotheses including: 1) metasomatic replacement of host andesite by hydrothermal fluids of either basinal or magmatic-hydrothermal derivation precipitating magnetite upon cooling;^16,22,25^ or 2) ascent of magmatic magnetite via flotation facilitated by attached fluid-solid aggregates with deposition due to decompression^20,23^.

We present the results of a high-resolution study of melt inclusions at the nanometer scale entrapped in plagioclase phenocrysts from three carefully selected andesite samples at the ELVC and located in the vicinity of the major MtAp orebodies (Fig. 1 and Supplementary Fig. S1). We present data on microtextures, chemical compositions, and phase proportions of crystallized immiscible melts preserved in melt inclusions, and also the temperature of melting of the solid phases, and the chemical composition of the parental melt. The results were obtained using high resolution-transmission electron microscopy (HR-TEM), a field emission gun-electron probe microanalyzer (FEG-EPMA), microthermometry, Raman spectroscopy, and EPMA (see Methods section). Our results reveal the unexpectedly complex nature of melt immiscibility during the magmatic evolution of the El Laco system, and illustrate how melt immiscibility drives MtAp mineralization.

## Results

### Immiscible melt inclusions textures and compositions

**Fig. 2:**
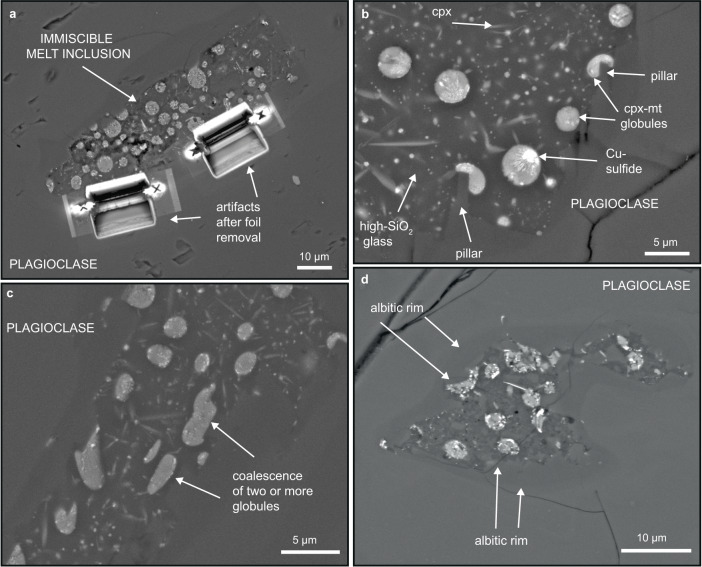
Morphology of immiscible melt inclusions in plagioclase; scanning electron microscope back-scattered electron images (SEM-BSE). **a** Melt inclusion with artifacts after removal of two transmission electron microscopy (TEM) foils. **b** Melt inclusion with immiscible clinopyroxene-magnetite globules (cpx-mt) and Cu-sulfide phase contained in high-SiO_2_ dacite glass with euhedral clinopyroxene (cpx) crystals. Destabilized growth face of albitic rim by attached cpx-mt globule resulting in formation of pillar structure^30^. **c** Coalescence of cpx-mt globules. **d** Arrows point to albitic rim in plagioclase phenocryst surrounding the melt inclusion.

**Fig. 3:**
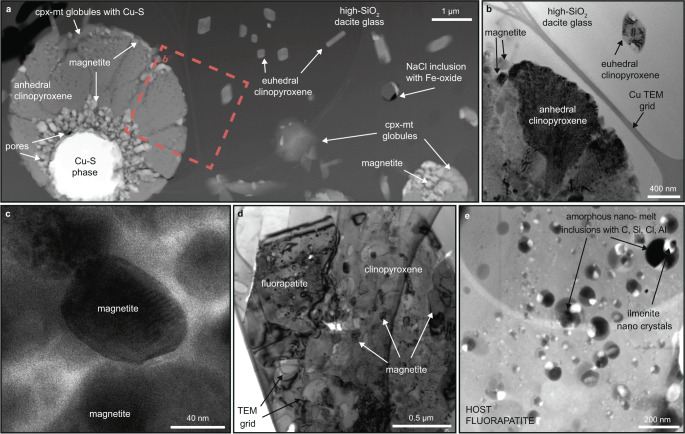
High resolution-transmission electron microscopy (HR-TEM) images of phases in melt inclusions. **a** High-angle annular dark field (HAADF) overview image of typical textural relation between conjugate Fe-rich and Si-rich melts: clinopyroxene-magnetite (cpx-mt) globules with occasional Cu-sulfide (digenite) globules embedded in high-SiO_2_ dacite glass with euhedral clinopyroxene crystals. **b** Bright field (BF) image of two generations of clinopyroxene crystals. **c** BF image of anhedral magnetite hosted in clinopyroxene from cpx-mt globules. **d** BF image of subhedral apatite in clinopyroxene-magnetite globule. **e** HAADF image of immiscible nano melt inclusions in host apatite from **d**.

**Fig. 4:**
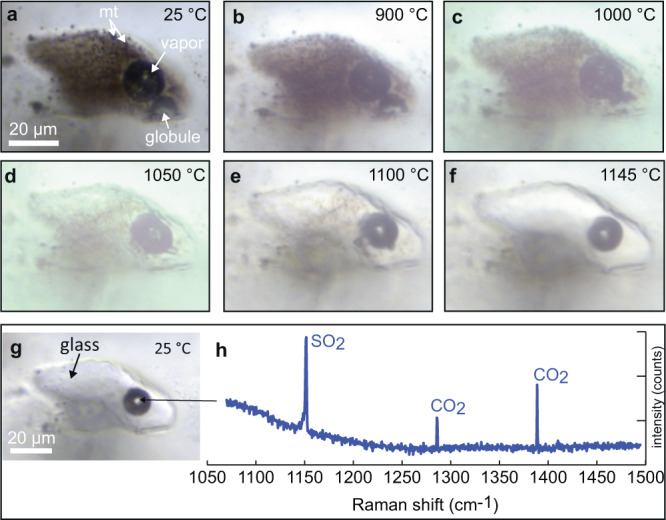
Heating sequence and Raman spectrum of the vapor phase in the homogenized melt inclusion. Temperature series of photomicrographs of a representative melt inclusion hosted in plagioclase in sample LCO-1. At room temperature prior to heating (**a**), the inclusion contains silicate glass as well as a vapor bubble, numerous sub-micron crystallites of magnetite (mt), and a globule of semi-opaque material. During heating (**b**–**f**), the globule and crystallites melt progressively at temperatures >900 °C and are fully dissolved in the liquid at 1145 °C (**f**) in all melt inclusions analyzed. After quenching (**g**), the inclusion contains only a clear, homogeneous glass plus vapor. **h** A representative Raman spectrum of the vapor phase in the inclusion after quenching, showing the characteristic peaks of SO_2_ vapor (~1151 cm^−1^) and the Fermi diad of CO_2_ (~1286 and 1389 cm^−1^).

**Fig. 5:**
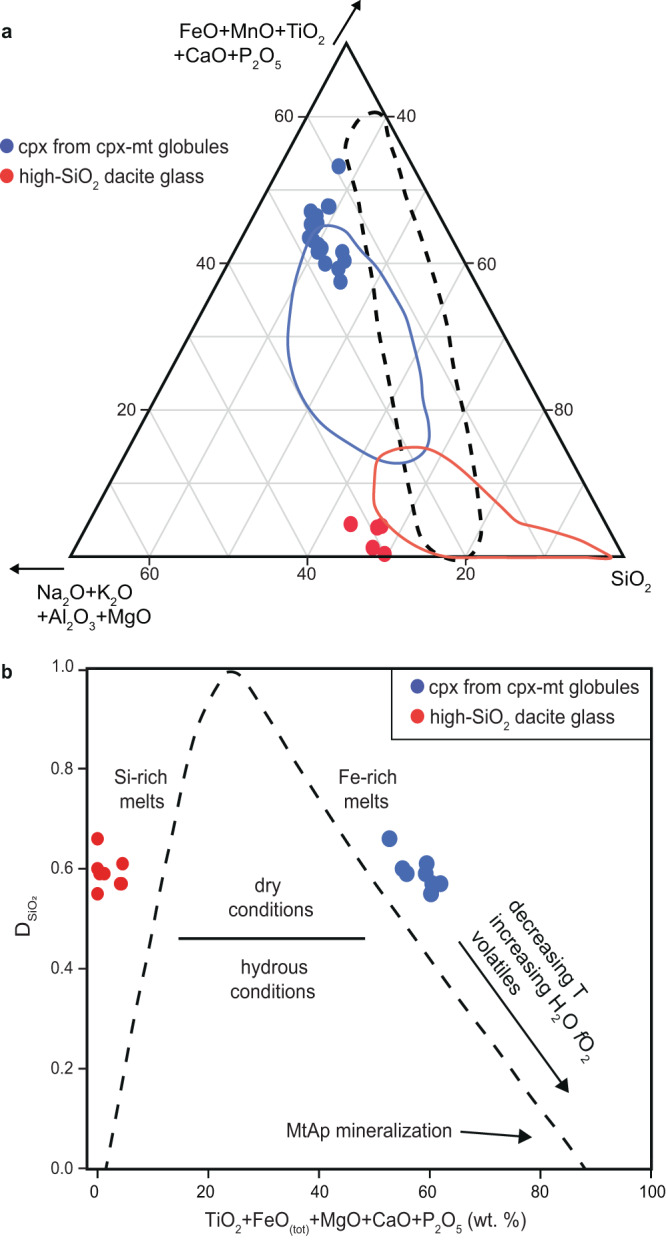
Chemical composition of Fe-rich and Si-rich melts. **a** Ternary plot showing immiscible Fe-rich and Si-rich melts^32^ based on field emission gun-electron probe microanalyzer (FEG-EPMA) point analyses of clinopyroxene (cpx) from clinopyroxene-magnetite (cpx-mt) globules and high-SiO_2_ dacite glass (Supplementary Table S2, S3). Note that Fe-rich apex records extremely complex immiscibility. Blue and red fields—previous chemical analyses of melt inclusions in El Laco andesite^6^. Immiscibility fields inside the dashed line were established experimentally^71^. **b** Plot of SiO_2_ partitioning between the Fe- and Si-rich conjugate melts as a function of elements entering the Fe-rich melt with plotted average composition of conjugate melts in individual melt inclusions from this study (after refs. ^49, 50^). The dashed line following the conjugate melt pair compositions is based on experimental data on intermediate-composition magmas and tholeiitic systems, and on natural immiscible melt globules^49, 50^. See also Supplementary Fig.S8.

**Fig. 6:**
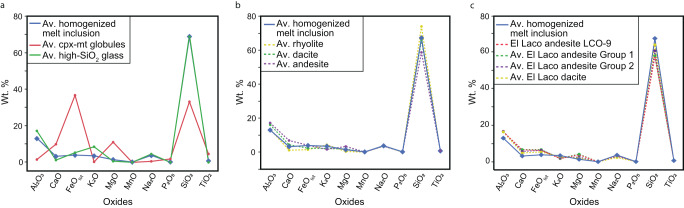
Composition of immiscible Fe-rich and Si-rich melts and parental melt. The comparison of average composition of homogenized melt inclusions (Supplementary Table S1) with: **a** clinopyroxene-magnetite (cpx-mt) globules and high-SiO_2_ dacite glass from crystallized melt inclusions; **b** examples of volcanic rocks;^72^ **c** average compositions of El Laco host rocks samples^7^ and whole rock LCO-9 sample hosting immiscible melt inclusions from this study (Supplementary Table S12). See also Supplementary Fig. S9.

The melt inclusions comprise two main contrasting solid phases; clinopyroxene (cpx) and magnetite (mt) globules (1–6 µm in diameter), which are enclosed in a high-Si, Al-K-Na dacite composition glass (Figs. 2, 3a, Supplementary Fig. S2), that also hosts a vapor bubble (Fig. 4). The melt inclusions are surrounded by an albitic rim on the host plagioclase that in some cases forms a pillar structure that deforms the adjacent cpx-mt globules and similar to those described by^30^ (Fig. 2b–d). The cpx-mt globules are interpreted to be the crystallized product of an Fe-rich melt (Fig. 5a) and are texturally similar to other natural samples recording Fe-rich and Si-rich immiscibility (e.g.^31^,). Phase proportions of the Fe-rich globules and high-SiO_2_ dacite glass are estimated, on average, at 14 modal % (range: 5–30%) and 86 modal % (range: 70–95%), respectively (Supplementary Table S5). The high-SiO_2_ dacite glass also includes ~5 modal % of euhedral clinopyroxene crystals having low Ti and negligible P contents based on SEM-EDS analysis with the HR-TEM (Supplementary Figs. S2d, S3h, S3i, S4d). The high-SiO_2_ dacite glass is partially devitrified and also hosts minor 150-300 nm globules of NaCl ± Fe-oxide (Fig. 3a, Supplementary Fig. S2d–f) and anhedral K-feldspar crystals. We calculated the average compositions of Fe-rich and Si-rich conjugate melts based on phase proportions and FEG-EPMA spot analyses on cpx from cpx-mt globules and high-Si glass without cpx phenocrysts (Supplementary Table S1–S4). The re-calculated composition of the Fe-rich melt from our melt inclusions is 40 wt.% SiO_2_, 24 wt.% FeO_tot_, 13 wt.% MgO, 12 wt.% CaO, 6 wt.% TiO_2_, 2 wt.% P_2_O_5_, 2 wt.% Al_2_O_3_, and <1 wt% of Na_2_O, K_2_O, and Cl. Conversely, the re-calculated Si-rich melt consist of 67 wt.% SiO_2_, 17 wt.% Al_2_O_3_, 9 wt.% K_2_O, 5 wt.% Na_2_O, 5 wt.% FeO_tot_, and <1 wt% of TiO_2_, CaO, MgO, P_2_O_5_ (Fig. 6a; Supplementary Table S1). The chemical compositions of Fe-rich globules and conjugate high-SiO_2_ dacite glass show visibly defined fields representing the immiscibility gap^32^ (Fig. 5a).

The spherical cpx-mt globules form a consistent phase assemblage of ~82 modal % clinopyroxene of augite-pigeonite composition with TiO_2_ and P_2_O_5_ contents of 5.59 and 2.26 wt.%, respectively, and ~18 modal % of magnetite (Supplementary Figs. S4c, S5; Supplementary Tables S1, S6). Additionally, the cpx-globules, in some cases, host Cu-S-rich globules and fluorapatite, which in turn hosts C-Si-Al-Cl-rich nano melt inclusions with ilmenite. The clinopyroxene in the cpx-mt globules is anhedral and shows a “cauliflower-like” texture, with irregular shapes (Fig. 3a, b). Two types of magnetite are hosted by clinopyroxene within the globules: 1) subhedral to anhedral, elongate crystals 50-200 nm in diameter with longer crystallographic axis oriented outward from the center of the globule (Fig. 3a, c); and 2) euhedral crystals 150-400 nm in diameter that have grown on the outer surfaces of the globules (Supplementary Fig. S2a, c). The cpx-mt globules contain pores, especially abundant in the polycrystalline magnetite (Fig. 3a).

Some of the cpx-mt globules contain inner “cores”, or nucleation sites, of subspherical 1–2 µm globules of predominantly Cu-S-rich composition (rarely Fe-Cu-S) that occur in some cases associated with nanoscale amorphous SiO_2_. The morphology of these Cu-S globules varies from spherical to subspherical, with a partially curved meniscus at the interface with the clinopyroxene (Fig. 3a). The TEM electron diffraction peak indexing of the Cu-S phase closely matches that of digenite (Cu_9_S_5_; Supplementary Fig. S3d,e). The FEG-EPMA analyses of the Cu-S show 64-74 wt.% Cu and 20-34 wt.% S, and the calculated stoichiometry indicates phase compositions between covellite (CuS) and digenite (Supplementary Tables S7,S8). The concentration of Cu in the cpx-mt globules (where the Cu-S phase is observed) is calculated to be as high as 10.6 wt.% with a Cu/Fe ratio of 0.32 as estimated from the volume proportions and chemical compositions (Supplementary Table S9, Supplementary Fig. S6).

Rarely, the cpx-mt globules contain <2 µm^2^ of subhedral fluorapatite that occupies ~5 modal % of the area of cpx-mt globules (Fig. 3d, Supplementary Fig. S3a; Supplementary Table S10). Larger euhedral and smaller anhedral magnetite crystals are associated solely with clinopyroxene and not fluorapatite. Both fluorapatite and clinopyroxene contain multiple solid inclusions having different compositions and crystal morphologies. The clinopyroxene contains Fe- and Ca-rich lamellae and hosts multiple inclusions of quartz. Several <250 nm, subhedral to euhedral, magnetite crystals with minor Ti, Mg, and Al are located at the edge of, or adjacent to, quartz inclusions (Supplementary Fig. S3c). The clinopyroxene in the cpx-mt globules also contain droplets of a putative phosphate-rich melt crystallized to fluorapatite. The fluorapatite hosts regularly distributed and abundant, <100 nm, spherical nanoscale melt inclusions composed of C-Si-Al-Cl-rich glass with daughter crystals of ilmenite with Th-Nb-Y-U-Zr (Fig. 3e). The composition of the glassy nano melt inclusions and daughter ilmenite were determined semi-quantitatively by SEM-EDS analyses with the HR-TEM (Supplementary Fig. S4g-h) and their structure was determined by high-resolution lattice fringe images with the calculated diffraction pattern (Supplementary Fig. S3l-m). The fluorapatite also hosts: (1) rare, individual Ca-phosphate REE-C-rich Si-F-S nanocrystals; (2) Si-O-Cl-Fe-Ti melt inclusions; and, (3) solid NaCl inclusions. The modal proportion of these nanoscale melt inclusions in the fluorapatite is ~13% (Supplementary Table S10). The presence of phosphate inclusions in the clinopyroxene and silicate inclusions in apatite suggests coeval, but separate, crystallization. To our knowledge, this is the first reporting ever of melt inclusions occurring within a crystal inside another melt inclusion, all at the nm scale.

### Melting temperature of immiscible phases

During the microthermometry heating experiments of the melt inclusions, the first onset of melting was observed between 900 and 1000°C in all inclusions (Fig. 4a–g). The opaque to semi-opaque globules (i.e., cpx-mt globules) progressively dissolved into the interstitial liquid, and the minute crystals/globules of magnetite dissolved progressively, such that the inclusions went from dark and cloudy at lower temperature, to clear and colorless at T > 1100 °C. Above 1100 °C, only a few small globules of opaque material remained, and these were fully dissolved at 1145 °C in almost all of the melt inclusions analyzed except for a few inclusions. In the few inclusions where a small opaque grain remained at this temperature, the remaining grain was always <1 volume% of the melt inclusion. The vapor bubble shrank in size somewhat at this temperature, but did not homogenize completely into the liquid. We did not attempt to heat further to dissolve the vapor bubble because the heating experiments were conducted at ambient external pressure (see^33^). After the inclusions had fully melted at 1145 °C, the chips were quenched instantaneously by removal from the furnace. In nearly all cases, this resulted in quenching to a homogeneous, colorless, glass showing that the immiscibility process recorded in the melt inclusions is reversable to a single-phase state as in any equilibrium process (Fig. 4g).

Raman analyses of the vapor bubbles within both unheated and quenched inclusions reveal the presence of both CO_2_ and SO_2_ in the vapor phase (Fig. 4h). Based on the splitting of the Fermi diad^34^, the density of CO_2_ in the bubbles is approximately 0.02 g/cm^3^. Based on the relative intensities of the Raman peaks of SO_2_ and CO_2_ (SO_2_ vapor at ~1151 cm^−1^, and CO_2_ at ~1286 cm^−1^ and 1389 cm^−1^), and the relative Raman scattering efficiencies of these two gases^35^ the vapor phase consists of approximately equal molar proportions of SO_2_ and CO_2_. To our knowledge, these vapor bubbles are the most SO_2_-rich vapor bubbles ever reported in a melt inclusion study (1:1 in our study vs. 1:84 in ref. ^36^).

Raman analyses of the glass show no peaks for H_2_O, i.e., it is below detection limit, suggesting that the H_2_O content of the andesitic glass is less than about 0.8 wt% H_2_O^37^.

### The composition of the primary melt

The average composition of the homogenized melt inclusions reaches 67 wt.% SiO_2_, 13 wt.% Al_2_O_3_, 4 wt.% FeO_tot_, Na_2_O, and K_2_O, 3 wt.% CaO, 2 wt.% MgO, and <1 wt.% TiO_2_, P_2_O_5_, MnO, and Cl with 270 ppm of F, 162 ppm of Cu, and 117 ppm of S (Fig. 6; Supplementary Fig. S7; Supplementary Tables S1, S11). The composition varies around the average composition of dacite, and is generally more felsic than the average compositions of andesite samples from the ELVC^7^, or the whole rock composition of the host andesite with plagioclase phenocrysts hosting the immiscible melt inclusions from this study (sample LCO-9; Fig. 6b-c; Supplementary Table S12).

## Discussion

The individual melt inclusions record a small-scale, complex and relatively early evolution of magma immiscibility by which the different melts could have separated from each other during cooling. Various solid phases in the melt inclusions have distinct compositions and curved menisci, i.e., the cpx-mt globules in high-SiO_2_ dacite glass, the Cu-S globules in cpx-mt globules, and the C-O-Si-Cl-Al-HFSE nanoscale melt inclusions in fluorapatite (Figs. 2, 3). The abundance of solid globules, their variable sizes within and among multiple melt inclusions, with consistent phase proportions and chemical compositions, strongly suggests that these solid droplets represent primary, immiscible melts^38^. Some large melt inclusions can have a slightly different chemistry from the primary melt due to interaction with the plagioclase phenocryst host^39^ and formation of an albitic rim surrounding the melt inclusions^30^, but this did not change the concentration of key elements in melt inclusions.

Multiple studies interpreted the presence of texturally and compositionally similar melt inclusions to those at El Laco as the evidence of melt immiscibility, e.g., immiscible melt inclusions obtained through the experiments (e.g.^40–43^,), discovered in the interstitial melts in lunar basalts (e.g.^44–46^,), or mesostasis of tholeiites (e.g.^31,47^,). Similar association of various immiscible liquids, i.e., silicate, Fe-oxide, and Cu–Au–Ag has been also recorded in microspherules of magnetite ore and pyroclastic sequences of the Kostenga iron deposit^48^. Thus, such numerous and heterogeneous entrapment of melt inclusions in our study suggest the presence of multiple, complex, interconnected, and immiscible liquids: Fe-rich, Si-rich, sulfide- rich, phosphate-rich, and C-O-Si-Cl-Al-HFSE-rich.

Our interpretation is that the magmatic system leading to the formation of the El Laco MtAp mineralization initiates with the crustal contamination of parental calc-alkaline melt^3^ that triggers the separation of conjugate Fe-rich (i.e., Fe-Mg-Ca-P-Ti-F-Cu-S-C-O-Cl-HFSE) and Si-rich (i.e., Si-Al-K-Na-O)^7^ at high temperature (>1145°C). Elemental distribution of these Fe- and Si-rich immiscible melts agrees, within limitations of analytical uncertainty, with the fractionation of elements between Fe- and Si-rich melts reported in the studies of both experimental and natural samples^41,49–51^ (Fig. 5).

After separation, the Fe-rich melt eventually crystallizes upon cooling as clinopyroxene and magnetite. The results from experiments have shown that Cu and S partition preferentially into the Fe-rich phase^51,52^, and that the addition of <3 wt.% of S into a silicate liquid of intermediate composition (±H_2_O, P, F) at 1000-1200°C stabilizes a third immiscible sulfide liquid^41,50^. In those experiments, the sulfide melt formed a sphere within the globule of immiscible Fe-rich melt embedded in the Si-rich melt^41^. Those textures and phase relationships are remarkably similar to those reported in the present study (Fig. 3a), and suggest that the formation of an immiscible sulfide liquid is closely related to the Fe-rich melt. Typical magmatic sulfides, however, have much lower Cu/Fe ratios (e.g., pyrrhotite, chalcopyrite) than the sulfide melt that crystallized in these melt inclusions (≤1 vs. >60, respectively). Experimental studies on those systems have shown that the last sulfide phase formed before S transition from the sulfide to sulfate state (at NNO + 1 *f*O_2_^53^) is a Cu-rich sulfide (e.g., chalcocite Cu_2_S) that can coexist in equilibrium with anhydrite at sub-magmatic temperatures^54^. The results from a recent study showed that an increase of redox buffer causes the transition from reduced S-rich immiscible melt to Ca-S-O melt in the presence of immiscible P-Fe-rich melt^42^. The presence of the magnetite and digenite assemblage in the El Laco melt inclusions strongly suggests that the system overall formed under high *f*O_2_–*f*S_2_ conditions, probably close to the hematite-magnetite and covellite-digenite phase boundaries^55^.

In our model, as the system cooled, a final phosphate-C-O-Si-Cl-Al-HFSE melt separated from the Fe-rich melt. Crystallization of fluorapatite then produced a final residual melt enriched in incompatible elements that is represented by the nanoscale melt inclusions containing the carbonates, ilmenite, halite and other chlorides, and minor SiO_2_. Droplets of a phosphate-rich melt (i.e., fluorapatite with curved menisci) in the silica-rich glass of the melt inclusions have previously been reported from pyroxene phenocrysts in the ELVC andesite^6^ and the presence of immiscible Fe-P-S-O melts has also been observed at El Laco in unconsolidated pyroclastic ore^13,56^. In intrusive MtAp systems, fluorapatite forms large massive bodies that typically cap the magnetite ore which supports its late crystallization^7,24^. Despite the lack of direct evidence for the presence of liquid water, the cpx-mt globules in melt inclusions contain multiple pores that suggests the presence of a low-density aqueous phase exsolved during cooling from the crystallizing melts^6^ (Fig. 3a).

We have not observed anhydrite within the El Laco melt inclusions; however, it has been described in similar melt inclusions in andesite from El Laco^6,14^. Still, very high concentration of SO_2_ was found in the vapor bubble within the melt inclusions, suggesting that sulfate was also a significant component in this magmatic system. This points to the high-temperature degassing processes that occurred at El Laco^7^. During our studies, we have not found individual vapor-rich fluid inclusions with only SO_2_ and other volatiles in plagioclase phenocrysts. This suggest that the melt was not actively degassing SO_2_ at the time of trapping; though, it was probably near saturated and may have begun degassing soon after and at slightly shallower depths. This is in agreement with the ^18^O range of compositions of acid-sulfate alteration at El Laco, which points out to the reaction between high-temperature magmatic fluids with meteoritic water^7^.

**Fig. 7:**
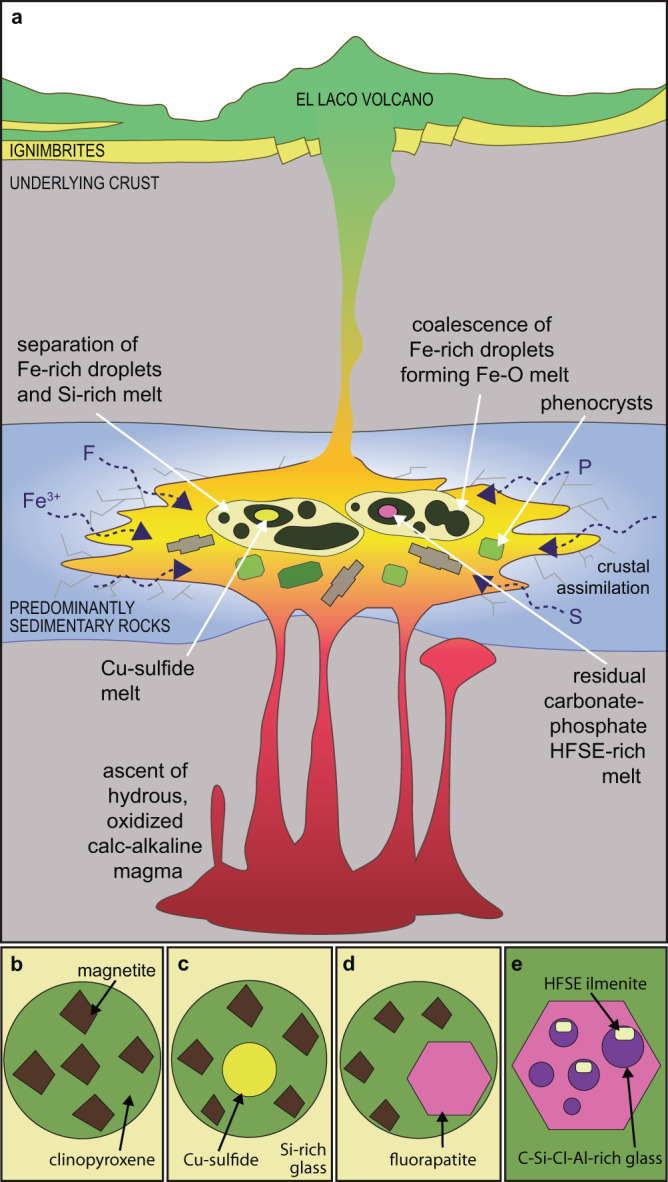
The genesis of the El Laco MtAp deposit. **a** Model of complex separation of multiple immiscible melts in the El Laco andesitic magma chamber. **b**–**d** Drawings of typical mineral phases in globules embedded in Si-rich dacitic glass hosted by immiscible melt inclusions found in this study. **e** Drawing of immiscible nano melt inclusions in fluorapatite from (d). Drawings are not to scale.

Our results point to a direct, parallel, relationship between the relatively early evolution of immiscible melts recorded by melt inclusions at the nanoscale, and the formation of MtAp deposits at the macroscale (Fig. 7). The dominant mineral assemblage is the same and includes magnetite, Mg-Ca-rich clinopyroxene, fluorapatite, and anhydrite. This phase assemblage is consistent with an early formation of a magnetite-rich body with later crystallization of clinopyroxene and fluorapatite, that can form pegmatite-like bodies composed of Ca-Mg silicates, fluorapatite (commonly with inclusions of monazite), and variable amounts of magnetite, ilmenite, and anhydrite^7,28^. The latter result is similar to a recent study^57^ that showed that apatite, actinolite, and magnetite from other MtAp ore bodies, i.e. at the Buena Vista (Nevada, USA) and Iron Springs deposits (Utah, USA), host carbonate-sulfate-Fe-rich melt inclusions resembling the final, residual melt. At El Laco, a Fe-sulfate-rich melt was also found in the melt inclusions from the late ore-stage diopside-magnetite-anhydrite veins^27^, which suggest that the residual melts at El Laco were increasingly enriched in sulfate components, leading to the final crystallization of large accumulations of massive anhydrite^7^. This is also consistent with the presence of Fe-P-S-O melts in a form of ejected fragments of destinezite (Fe_2_(PO_4_)(SO_4_)(OH)·6H_2_O; aka diadochite in well crystallized forms, found in pyroclastic bombs and in the unconsolidated hematite-rich epiclastic sediments at El Laco^13,15,56^ and with experiments that report phosphate-rich immiscible melts that had separated from an Fe-rich melt^13,42^.

We acknowledge that the cpx-mt globules, representing the Fe-rich melt in our melt inclusions, have high concentrations of SiO_2_, i.e., av. 40 wt.% (Supplementary Table S1) in comparison to the low-SiO_2_ concentrations in the MtAp ore. Our interpretation is that the immiscible melts were trapped in plagioclase too early, and at a very high temperature (>1145° C), to be able to reach the extremely contrasted compositions of immiscible melt endmembers having ${{{\mathrm{D}}}_{{{\mathrm{SiO}}}_{2}}}$^LFe/LSi^ values well below 0.2 (Fig. 5b). The average 40 wt.% SiO_2_ of the cpx-mt globules indicate high values of partition coefficient D, that describes SiO_2_ partitioning between conjugate Fe-rich and Si-rich melts (av. 0.59 ${{{\mathrm{D}}}_{{{\mathrm{SiO}}}_{2}}}$^LFe/LSi^; Fig. 5b; (see^49,50^). This suggests that if the immiscible system can cool significantly, then more compositionally extreme melts form; but that only happens when melts are rich in P, F, or H_2_O^49,50^. Moreover, a simple geochemical approach suggests that if the immiscible melts become increasingly contrasted in composition with cooling, most of the Si^4+^ from the Fe-rich melt would partition into the Si-rich melt. The increasing amount of Fe^2+^/Fe^3+^ would then be used to form magnetite, whereas leftover minor Si^4+^, Ca^2+^, and Mg^2+^ would form diopside and Ca^2+^, P^5+^, and F^-^ would form apatite, like what is found in the MtAp ore.

We advocate that the key process that initiated the formation of the Si-depleted, Fe-P-S-C-O-HFSE-rich melt, key to forming MtAp mineralization, is crustal assimilation by the ascending andesite. The host ELVC andesite has highly radiogenic Sr isotopes values^7^ indicating significant interaction of primitive melts with crustal components^3,5^. The MtAp ore has higher radiogenic Sr crustal values (^87^Sr/^86^Sr 0.7083) than the interbedded andesite (^87^Sr/^86^Sr 0.7066–0.7074), which has been interpreted as reflecting varying degrees of assimilation of Nd-poor continental rocks by the primitive melts at depths greater than 1–2 km^7^. Moreover, the Pb-Pb isotope values from whole-rock study of El Laco andesite lie between the orogenic and upper-crust evolution curves^58^ suggesting involvement of crustal component(s) in the system^8^. Potential crustal contaminants include evaporites and Fe-Mn-rich carbonates of the underlying Salta Group, Yacoraite Formation^7,59,60^ of which skarn xenoliths have been reported in the calc-alkaline magmas of the Lascar volcano located ~50 km N from El Laco^59^, and/or P-rich oolitic ironstone and coquinas of Ordovician-Devonian age^7,61,62^ that underlie the ELVC. The presence of NaCl ± Fe oxide globules in the high-Si glass in our melt inclusions support the involvement of crustal contamination, i.e., evaporites, in the formation of immiscible melts^27^. Experimental studies and phase equilibria modeling^13,42^ have also shown, that the elevated P, S, and F, concentrations, and an oxidized environment (higher *f*O_2_), expand the miscibility gap for Fe-rich and Si-rich melts^41^.

As stated in a recent study^7^ and confirmed by the disequilibrium between Pb-Pb isotope signatures of immiscible melt inclusions and El Laco magnetite ore (i.e., 18.61-19.07 ^206^Pb/^204^Pb vs. 18.22-18.51 ^206^Pb/^204^Pb;^8^), the clinopyroxene-magnetite globules are not strictly the parental melt from which the El Laco magnetite ore crystallized, but their poorly-contaminated earlier analogue. Only magmas that experienced a large degree of crustal contamination and incorporated enough amounts of phosphorus and fluorine can lower the solidus temperature, attain ${{{\mathrm{D}}}_{{{\mathrm{SiO}}}_{2}}}$^LFe/LSi^ values below 0.2-0.3 and form the Fe-rich low-SiO_2_ melts that can form the MtAp ore. Only the latter, with high Fe/Si ratios, have a viscosity low enough to be able to coalesce, ascend, and form MtAp mineralization like that found at El Laco, with the Fe-rich melt ascending along tensional or collapse-related fractures^63^.

Furthermore, the results from our study have implications beyond the genesis of MtAp mineralization. The formation of Fe-O melts that later form massive magnetite deposits inhibit the formation of Fe-Cu-S melts, as Cu sequesters the small amounts of available reduced sulfur. In less oxidized calc-alkaline arc magmas (*f*O_2_ < NNO + 1), any Cu stored in the immiscible Cu-sulfide melt could lead to the formation of Cu-mineralization. The presence of Cu-S in these melt inclusions opens the possibility for genetic links between these MtAp systems and other copper-rich deposits. Interestingly, one hundred kilometers southeast of El Laco, broadly coeval highly oxidized Cu-Au porphyry systems contain Cu-rich sulfides with this Cu-rich precursor and its further precipitation in a magmatic-hydrothermal system in a process similar to that described by^64^ at the slightly older (~7 Ma) Bajo de Alumbrera porphyry copper deposit. The Cu contained in the immiscible sulfide melt could supply this metal for coeval or later superimposed iron oxide-copper-gold (IOCG) mineralization. The results from our micro- and nano-scale study shows that the composition of the melt inclusions at El Laco resemble the mineral assemblage of the ore, suggesting a direct link between them. The composition of the melt inclusions also indicates that there are multiple interconnected immiscible melts but the dominant Fe-rich melt is able to produce large-scale MtAp mineralization. The similarities of the El Laco system with other MtAp deposits elsewhere suggests that analogous processes can lead to the formation of MtAp mineralization worldwide. The formation of these systems needs interaction of primitive melts with P-F-rich rocks, which seems the critical pre-requisite for the formation of these systems.

## Methods

### Materials

The samples of unaltered andesite hosting melt inclusions used in this study (LCO-1, LCO-9, and LAC-AND) were collected from Laco Sur, the east flanks of the ELVC, and north from Laco Norte (see Fig. 1b for sample location). LCO-1 sample is an unaltered andesite collected below the Laco Sur orebody. LCO-9 sample is unaltered massive andesite collected from the east slopes of El Laco summit, further from the orebodies. LAC-AND sample was collected from banded, poorly vesicular andesite flow, with little alteration, located above Laco Norte ore body. The andesite from all three samples includes 0.5-2 mm euhedral phenocrysts of plagioclase (45-54 modal%), orthopyroxene (~7 modal%), clinopyroxene (6-7 modal%), Fe-rich amphibole (~1 modal%), and titanomagnetite (~2 modal%). The groundmass in all three samples is composed of microliths of plagioclase, clinopyroxene, and titanomagnetite (28-35 modal%). The provided modal compositions are based on mineral liberation analyses (MLA)^8^ also available in Supplementary Information (Supplementary Fig. S10). The plagioclase phenocrysts occur as two populations. The first population includes large (1-3 mm), usually elongated, euhedral sieve-texture crystals or polycrystals with overgrown rims (Supplementary Fig. S1). The common resorption zones are filled with abundant melt inclusions exhibiting texture reflecting the coexistence of two immiscible melts (Fig. 2). The plagioclase phenocrysts composition varies from An_55_ to An_79_^6^. The second plagioclase phenocryst population includes small (100–500 µm) clear, unresorbed, euhedral crystals with normal growth zoning of composition between An_52_ and An_68_^6^ with no melt inclusions. Ortho- and clinopyroxene phenocrysts commonly form glomeroporphyritic aggregates with titanomagnetite and exsolved ilmenite. The pyroxene composition includes predominantly enstatite, diopside and augite^6^. Pyroxene phenocrysts host silicate melt inclusions with large shrinkage bubbles and daughter magnetite and apatite crystals, rarely silicates or anhydrite^6^. Amphibole phenocrysts are represented by rare, subhedral, resorbed hornblende crystals. Titanomagnetite microphenocrysts are usually associated with pyroxene crystals. They sometimes include exsolved ilmenite and apatite inclusions.

Melt inclusions hosted by plagioclase phenocrysts chosen for this study (Supplementary Fig. S1) show evidence for immiscibility between Fe-rich and Si-rich melts – Fe-rich spherical globules entrained in high-SiO_2_ dacite glass - Type 1 melt inclusions, based on categorization by^6^. For more information on other melt inclusion types as well as detailed petrographic study of plagioclase and andesite hosting immiscible melt inclusions, the reader is referred to the aforementioned study. Thin sections were investigated using an Olympus BX-50 transmitted light microscope at Memorial University of Newfoundland (MUN), St. John’s, Canada. The melt inclusions chosen were fully enclosed in the growth zones of the host phenocryst with no signs of post-entrapment leakage^38,65^. Thin sections were further analyzed using a JEOL-JSM 7100 F field emission gun scanning electron microscope (FEG-SEM) with back-scattered electron (BSE) imaging capabilities under a 15 kV rating voltage at MUN.

### High-Resolution-Transmission electron microscope (HR-TEM)

For the HR-TEM investigation, foils with approximately 15 × 7 x 0.15 μm dimensions were cut from the thin sections of the three samples mentioned above by focused ion beam (FIB) milling under ultra-high vacuum conditions using a FEI FIB200 instrument at the GeoForschungsZentrum Potsdam. The extracted foils were placed on a perforated copper grid, and analyzed using an FEI Tecnai G2 F20 X-Twin TEM at the GeoForschungsZentrum (GFZ) Helmholtz Centre, Potsdam, Germany.

The TEM was operated at 200 keV with a field emission gun as the electron source. High-angle annular dark-field (HAADF) images were acquired as Z-contrast images (camera length 75 mm) or Z-contrast + diffraction contrast images (camera length 220 mm) using a Fishione detector system. Bright- and dark-field images as well as high-resolution lattice fringe images were acquired as energy filtered images applying a 20 eV window to the primary electron beam. The system used was a Gatan Tridiem energy filter (GIF). Electron energy loss (EEL) spectra were acquired in diffraction mode. Ten spectra were acquired with an acquisition time of 1 second each. Analytical electron microscopy (AEM) was done using an EDAX X-ray analyser with an ultrathin window. The spectra were acquired in the scanning transmission mode scanning the electron beam within a preselected window thus minimizing mass loss during electron sputtering. The AEM acquisition time was usually for 60 seconds.

### Field emission gun electron probe micro-analyzer (FEG-EPMA)

Melt inclusions that contain the largest cpx-mt globules with the least number of magnetite crystals, and areas within the high-SiO_2_ dacite glass not compromised by any daughter crystals, were chosen for in situ FEG-EPMA compositional point analyses.

The FEG-EPMA analyses were acquired using a JEOL JXA-8530F Field Emission Gun Electron Microprobe (FEG-EPMA) using five wavelength dispersive spectometers (WDS) located in the Advanced Instrumentation Laboratory at the University of Alaska-Fairbanks. Energy dispersive X-ray spectra (EDS) were collected simultaneously and used to eliminate analyses where the beam spread into adjacent grains. Data were collected and reduced using Probe for EPMA v12.5.9 (probesoftware.com). Mass absorption corrections were made using the attenuation tables^66^.

For silicates, X-rays were collected using a 7 keV 1 nA focused electron beam. We estimate beam interaction volumes of approximately 900 nm (H) by 600 nm (V) based on Monte-Carlo simulations using CASINO (v2.51)^67^. Element migration was severe in spite of using an extremely low beam current and counts were corrected using a linear time-dependent intensity correction function in Probe for EPMA v12.5.9 software. Iron counts were collected from the Fe Ll-η line^68^ and backgrounds were modeled as polynomials. The chosen phases were small for the interaction volume which resulted in variable totals (see Supplementary Text in Supplementary Information file: Comments on FEG-EPMA totals). Standards analyses and conditions of analyses are included in Supplementary Table S4.

For copper sulfides, X-rays were collected using a 5 keV 3 nA focused beam. We estimate beam interaction volumes of approximately 300 nm (H) by 200 nm (V) based on Monte-Carlo simulations using CASINO (v2.51). Element migration did not appear to be a problem during the sulfide analyses. Calibration and reference standards of analyses are in Supplementary Table S8.

### Modal percentage and volume calculations of different phases in melt inclusions

To quantify phase proportions of (1) clinopyroxene-magnetite globules (cpx-mt) and high-SiO_2_ dacite glass in melt inclusions, (2) magnetite crystals and clinopyroxene in cpx-mt globules, (3) nano-melt inclusions and its host apatite, and (4) Cu-S mineral phases in cpx-mt globules, 10 images each (TEM and BSE images) were selected and analyzed with ImageJ 1.52a^69^. The results of all analyses are reported in Supplementary Tables S5, S6, S9, and S10, respectively.

The given protocol for all conducted image analyses follows an example of Cu_-_S volume calculations in cpx-mt globules from sample LCO-1_#4796 (Supplementary Fig. S6; Supplementary Table S9).

ImageJ 1.52a software allows for the segmentation of the areas of a digital image based on grayscale intensity, converted to a binary image (Supplementary Fig. S6), and then the calculation of the area occupied by the chosen phase(s).

The area of each phase calculated using ImageJ 1.52a can be represented as a perfect circle. Therefore, the radius of the assumed perfect circle is back-calculated based on the basic formula for the area of a circle. Having the radius of the whole globules and Cu-sulfide, the formula for the volume (V) of the sphere is calculated. The fraction of Cu-sulfide that occupies the whole globules is calculated based on the ratio of Cu-sulfide volume (V_CuS_) to whole globules volume (V_WG_) as below for example (sample LCO-1_#4796; Supplementary Table S9):

1 $$\%\, {{{{{\rm{Cu\; sulfide}}}}}}=\frac{{V}_{{{{{{\rm{CuS}}}}}}}}{{V}_{{{{{{\rm{WG}}}}}}}} \,*\, 100$$

2 $$\%\, {{{{{\rm{Cu\; sulfide}}}}}}=\frac{2.91}{47.32} \,*\, 100=6.15\%$$

### Microthermometry

Melt inclusions in all three samples were composed of silicate glass, opaque to semi-opaque globules, and sub-micron crystals of magnetite plus a vapor bubble at room temperature. Many of the larger inclusion showed somewhat variable phase proportions especially in terms of volume fractions of vapor and opaque globules; the latter inclusions generally showed signs of necking, and we interpret the variability of phase ratios as a result of necking after the onset of immiscibility. In contrast, the smaller melt inclusions showed remarkably consistent phase assemblage and phase ratios between all three samples.

For the homogenization heating experiments, doubly-polished wafers (30–150 µm) were prepared from samples LCO-1, LCO-9, and LCO-AND. Phenocrysts of plagioclase and diopside were inspected under transmitted light to identify melt inclusions. Plagioclase phenocrysts in all three samples contained abundant melt inclusions that ranged in size from ~10 to >100 µm. During the subsequent heating experiments, we mainly observed the smaller inclusions, as these were more commonly fully enclosed within the plagioclase host.

Heating experiments were done using a Linkam TS1400XY heating stage mounted on a custom Olympus BX53 microscope at University of Alberta, Canada. We used an initial heating rate of 100°C/minute up to 900°C, and a heating rate of 20°C/minute thereafter until the temperature of last melting was reached. We did not attempt to heat further to dissolve the vapor bubble because the heating experiments were conducted at ambient external pressure.

Raman analyses of melt inclusions and their contained vapor bubbles were done using a 532-nm laser and a Horiba LabRam HR Evolution Raman microscope at University of Alberta, Canada. Laser power was 100 mW at the source, and focusing was done using a 100x objective lens. Spectra of the vapor bubbles were collected using 60 seconds acquisition time and three accumulations.

### Electron probe micro-analyzer (EPMA) of homogenized melt inclusions

Quenched, glassy inclusions were exposed to the surface by gentle polishing. Subsequently, they were carbon coated and investigated using a JEOL 7100F field emission scanning electron microscope (SEM) with back-scattered electron (BSE) imaging capabilities at 15 kV at MUN. The major and trace elements of homogenized melt inclusions were measured using a JEOL JXA-8230 SuperProbe electron probe microanalyzer (EPMA) at MUN by calculating the average of compositional traverses crossing the melt inclusions. Traverse started in host plagioclase, went through albitic rim, melt inclusion, the albitic rim and ended in host plagioclase (Fig. S7). This methodology was chosen to be able to reliably distinguish the composition of melt inclusions from albitic rim and host plagioclase. Twenty seven melt inclusion from fourteen plagioclase in three samples were analyzed with nineteen melt inclusions having reliable compositions.

The EPMA glass analyses followed the modified methodology from ref. ^70^. Each of the traverses across the melt inclusions were analyzed two times using two separate element packages as different EPMA conditions were required. The first package included eight major elements: Na, Mg, Al, Si, K, Ca, Mn, and Fe, and accelerating voltage of 15 kV, 2 nA beam current, and 8 µm beam diameter. The second pass on the same traverses across melt inclusions included trace elements: F, P, S, Cl, Ti, and Cu and accelerating voltage of 15 kV, 140 nA beam current, and 8 µm beam diameter. The average composition of major elements in traverse across the melt inclusion were used to correct trace elements using ZAF techniques with the JEOL software. Kaersutite, apatite, pyrite, and cuprite standards were analyzed between unknown major and trace elements analyses.

### Supplementary information

Supplementary Information

## Data Availability

All data generated or analysed during this study, including the source data, are included in this published article and its Supplementary Information file. Geological samples were collected in a responsible manner and in accordance with relevant permits and local laws.
