# Supplementary material for: Magmatic immiscibility and the origin of magnetite-(apatite) iron deposits
Source: Nat Commun. 2023 Dec 19;14:8424. doi: 10.1038/s41467-023-43655-8 (PMC10730833; doi:10.1038/s41467-023-43655-8)
Supplement: Supplementary file 1 — Supplementary Information [file 41467_2023_43655_MOESM1_ESM.pdf]

Supplementary Information

Magmatic immiscibility and the origin of magnetite-(apatite) iron deposits

Dorota K. Pietruszka[1], John M. Hanchar[1*], Fernando Tornos[1,2], Richard Wirth[3], Nathan A. Graham[4], Kenneth P. Severin[4], Francisco Velasco[5], Matthew Steele-MacInnis[6], Wyatt M. Bain[6,7]

[1]Department of Earth Sciences, Memorial University of Newfoundland; St. John's, NL A1B 3X5 Canada.

[2]Instituto de Geociencias (CSIC-UCM); Severo Ochoa 7, 28040 Madrid, Spain.

[3]GFZ German Research Centre for Geosciences, Section 3.5 Interface Geochemistry; Telegrafenberg, Potsdam 14473, Germany.

[4]Department of Geosciences, University of Alaska Fairbanks; Fairbanks, AK 99775, USA.

[5]Departamento de Mineralogía y Petrología, Universidad del País Vasco UPV/EHU; 48080 Bilbao, Spain.

[6]Department of Earth & Atmospheric Sciences, University of Alberta; Edmonton, AB T6G2E3, Canada.

[7]British Columbia Geological Survey, Ministry of Energy, Mines, and Low Carbon Innovation; Victoria, BC V8T, Canada.

[*]Correspondence to: jhanchar@mun.ca

Supplementary text

Comment on FEG-EPMA totals

There are no universally accepted values for “what total indicates a good electron probe microanalysis (EPMA) analysis” although typically values between 99% and 101% are considered “acceptable”, or “good”.” This is in spite of the fact that any total over 100% is physically impossible. The calculation of concentrations of particular analytes is subject to a variety factors (e.g., choice of standards, data reduction method, choice of mass absorption coefficients) and gets even more complex when all elements are not measured (for silicates, typically O, which makes up close to 50% of the total) but are added by some assumed (typically stoichiometric) method. While it is possible to calculate various analytical errors associated with an analysis[1,2] this is rarely rigorously done.

Nonetheless, common practice in the EPMA community is to strive for analytical totals in the 99-101% range, recognizing that there are problems when working with non-ideal samples, which include rough surfaces on samples, areas at the edges of grains where they may be slight “rounding”, or grains that are smaller than the interaction volume of the electron beam in the sample.

A field emission gun (FEG)-EPMA (as used here) is capable of creating a beam that is nanometers in width at the surface of the sample. However, the beam spreads when it enters the sample limiting the size of the volume that can be analyzed. Reducing the accelerating voltage (as we did here) reduces the size of the interaction volume, but also limits X-ray production. Attempts to increase X-ray production by increasing beam current produced element migration; indeed element migration was a problem in silicates even with traditionally normal beam currents, most likely because of the greatly reduced volume of interaction. Similar problems were found when increasing counting times; elements migrated in and out of the sample in rather unpredictable ways. The end result was that we obtained fewer X-ray counts than we would have liked, and the resulting analytical spread in the data is greater than for more traditional EPMA W or LaB_6 EPMA analyses. The choice of cut off values of 96-104% is to some extent arbitrary, but is based on the observation that within this range the measured atomic fractions within each sample type is relatively stable.

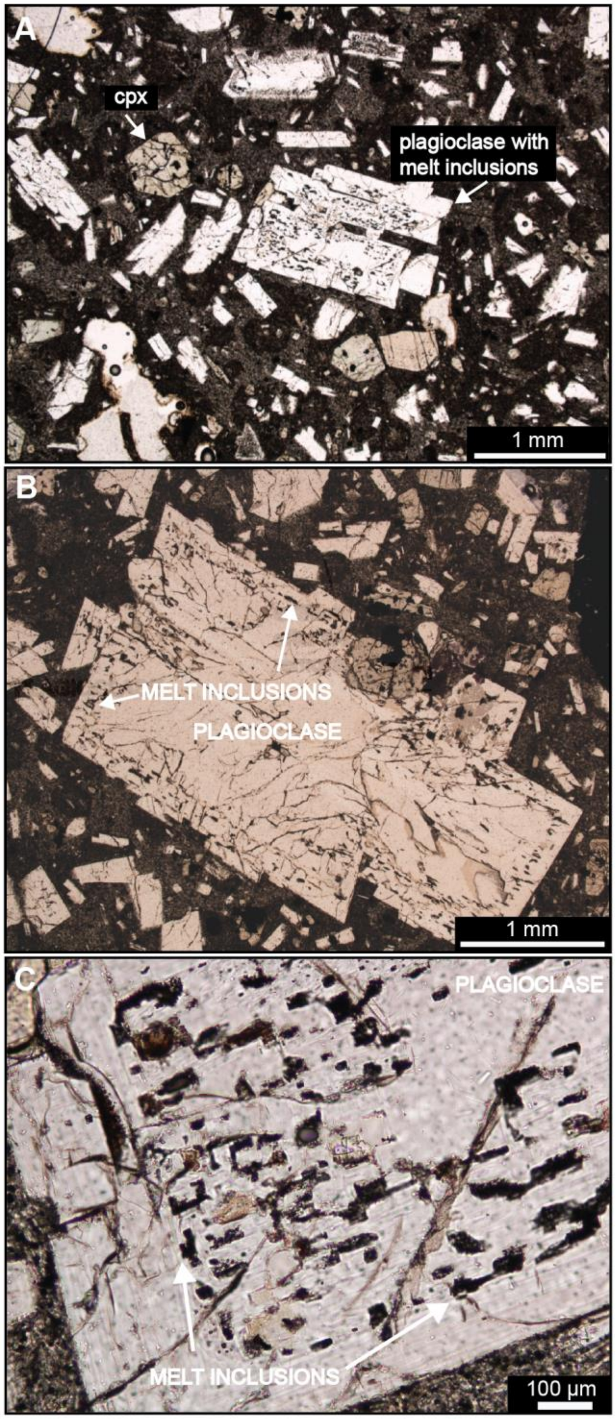

Fig. S1. Transmitted light images of host andesite and plagioclase phenocrysts hosting melt inclusions from this study.
(**A**) Overview of host andesite with plagioclase and minor clinopyroxene phenocrysts. (**B-C**) Sieve-textured plagioclase phenocrysts with overgrown rims. Resorbed zones contain abundant melt inclusions recording immiscibility between Fe-rich and Si-rich melts. Sample LCO-9.

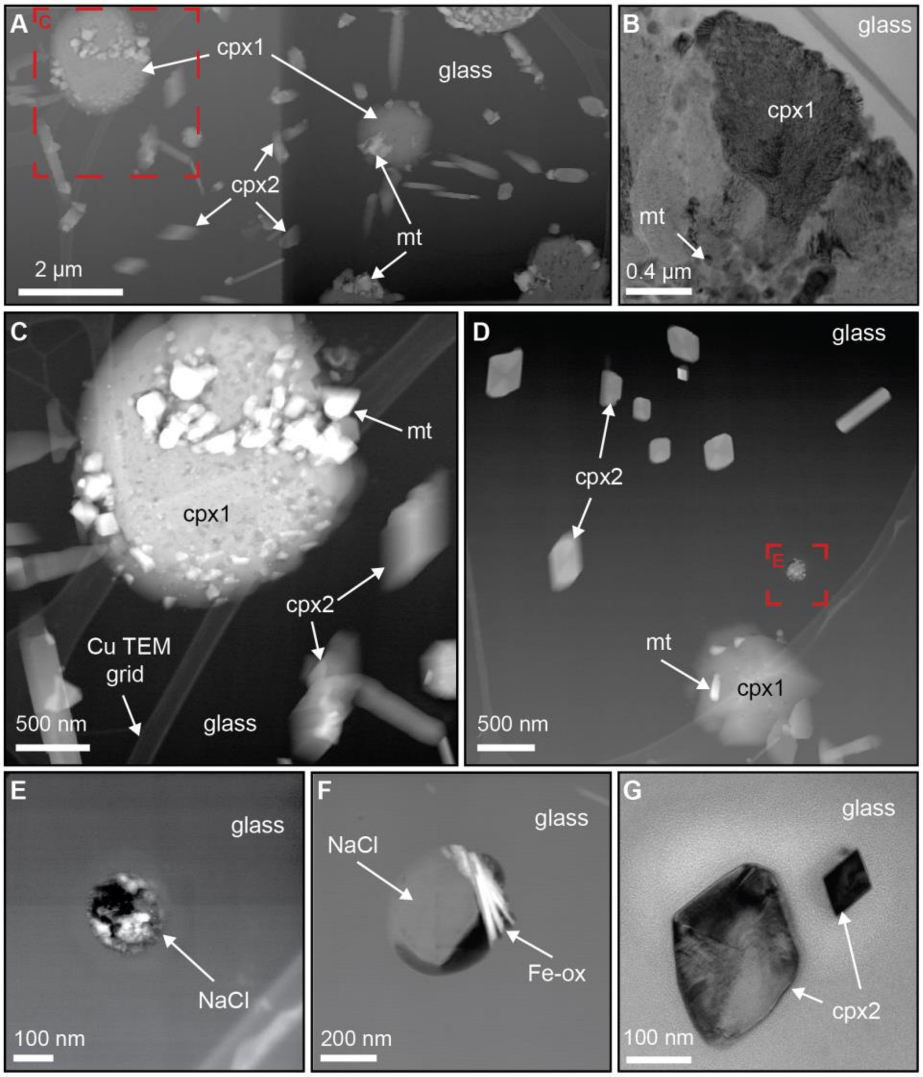

Fig. S2. Additional TEM images showing textures and mineralogical assemblage in immiscible melt inclusions.

(**A**) HAADF overview image of typical cpx-mt globules in this study. Note two morphologies of clinopyroxene - anhedral crystals in the cpx-mt globules (cpx1) and euhedral clinopyroxene crystals (cpx2) in the high-SiO_2 dacite glass. (**B**) BF image of cpx-mt globules with magnetite (mt) nano-crystals. (**C**) Close-up HAADF image in A of a cpx-mt globule comprised of cpx1 with euhedral mt crystals precipitated on the surface of the globule. (**D**) HAADF image of cpx1 and cpx2. (**E**) HAADF image of an inclusion composed of NaCl crystals in high-SiO_2 dacite glass. (**F**) HAADF image of inclusion of NaCl and Fe-oxide (Fe-ox) crystals, likely hematite, in high-SiO_2 dacite glass. (**G**) BF image of euhedral cpx2 crystals in high-SiO_2 dacite glass.

Dashed red rectangles in (A) and (D) above indicate location of images C and F. Abbreviations: BF image - bright field image, HAADF – high-angle annular dark field image. A and C images from TEM foil #4798; B, D-G images from TEM foil #4796.

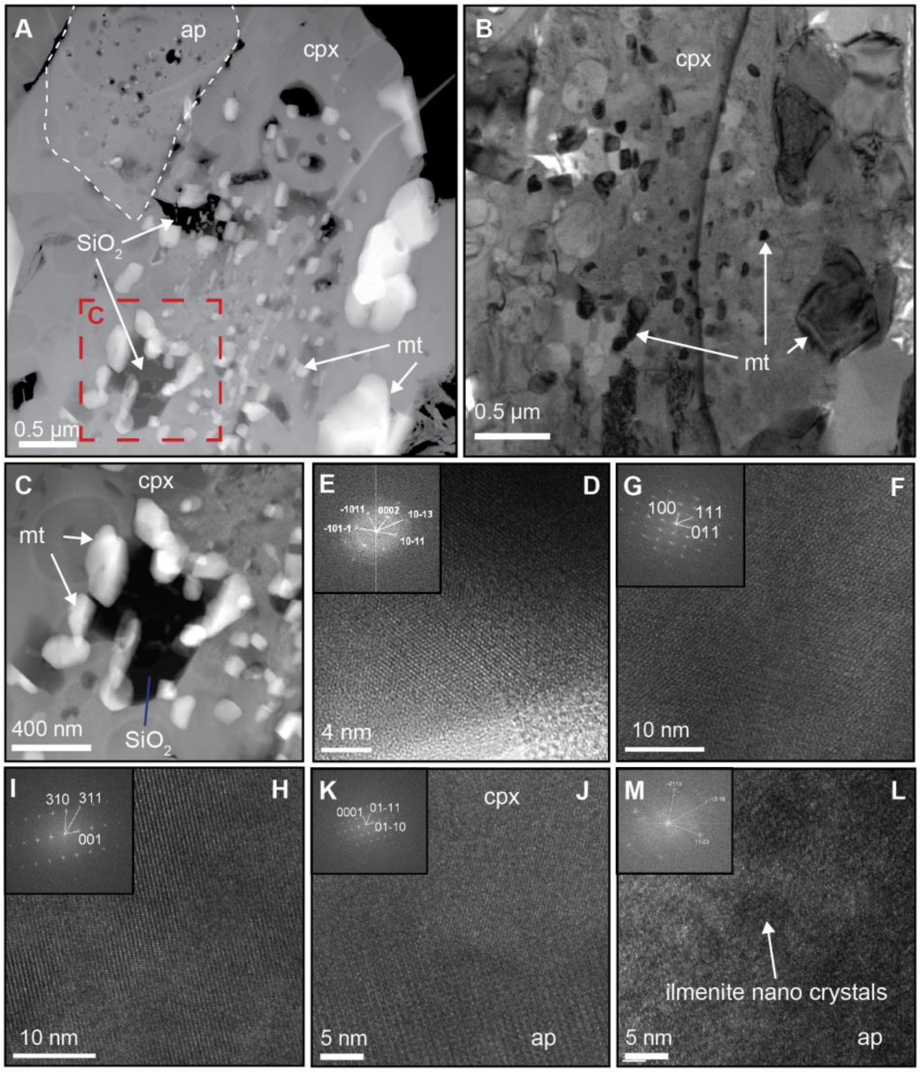

Fig. S3. Additional TEM, High Resolution-TEM (HR-TEM), and FFT images showing textures and mineralogical assemblage in immiscible melt inclusions.

(**A**) HAADF image of subhedral apatite (ap) and anhedral clinopyroxene (cpx) with inclusions of SiO_2 and magnetite crystals (mt) in a cpx-mt globule. (**B**) BF image of two types of mt: anhedral nano-inclusions and larger euhedral crystals. (**C**) Quartz in cpx outlined by mt euhedral crystals. (E-L) are high-resolution lattice fringe images with the calculated diffraction pattern FFT (Fast Fourier Transform) (E, G, I, K, M) inserted and indexed. HRTEM image of (**D**) Digenite, (**F**) Mt nanocrystal, (**H**) Cpx in high-SiO_2 dacite glass, (**J**) Apatite interface with clinopyroxene, (**L**) Ilmenite daughter crystal in amorphous nano melt inclusions hosted by apatite from J. FFT indexed of: (**E**) Digenite, (**G**) Mt nanocrystal, (**I**) Cpx in high-SiO_2 dacite glass, (**K**) Apatite, and (**M**) Ilmenite daughter crystal.

Abbreviations: BF image - bright field image, HRTEM - high resolution TEM, HAADF – high-angle annular dark field image, FFT - Fast Fourier Transform. All images from foil #4800.

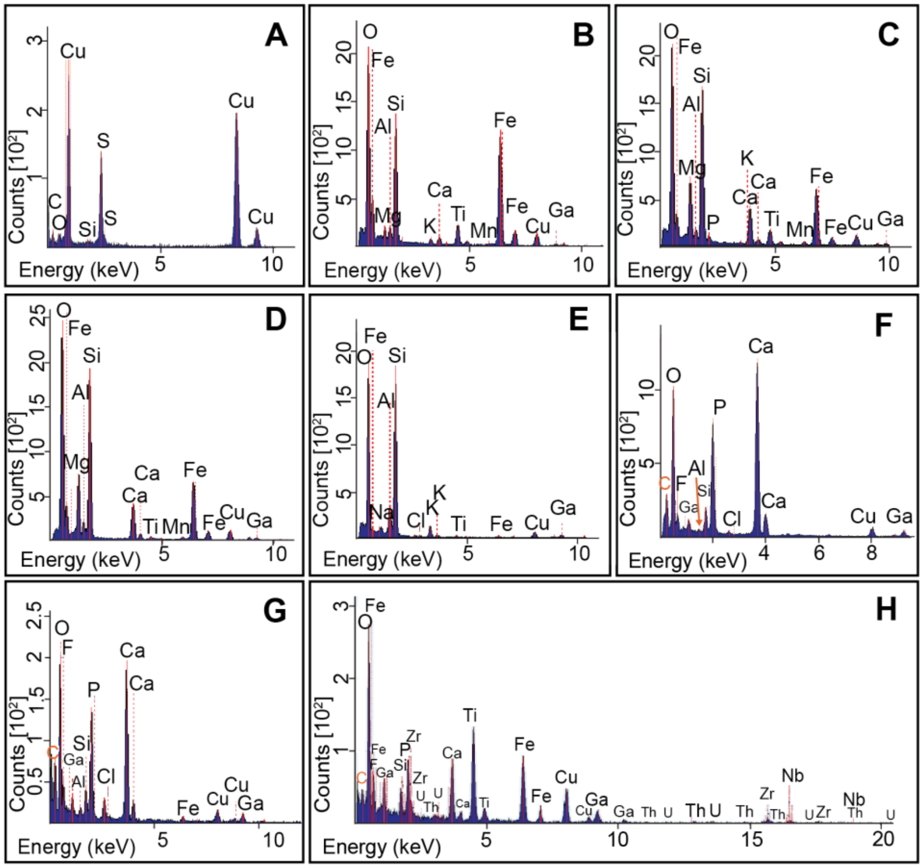

Fig. S4. TEM -EDS analyses of selected phases.

(**A**) Digenite; (**B**) Magnetite nanocrystal in cpx-mt globules; (**C**) Clinopyroxene from cpx-mt globules; (**D**) Euhedral clinopyroxene needles in high-SiO_2 dacite glass, (**E**) High-SiO_2 dacite glass. (**F**) Fluorapatite in cpx-mt globules. (**G**) Amorphous nano melt inclusions hosted by apatite analyzed in (F); Ca, P, O, F are most likely coming from host apatite. (**H**) Ilmenite daughter crystals in nano melt inclusions hosted by apatite analyzed in (F); Ca, P, F, S, and Si peaks are most likely coming from the amorphous nano melt inclusions and host apatite; HR-TEM and FFT of ilmenite in Figure S3, M and L. Based on the G and H spectra the composition of the residual melt, i.e., C-O-Si-Cl-Al-HFSE-rich melt, follows the highest to lowest peak counts of glassy nano melt inclusion and daughter ilmenite, discounting the elements that most likely come from the host phases.

Notes: small Cu and Ga peaks in the analyses reflect the composition of Cu TEM grid, and the residual Ga that was used to extract the foils with the focused ion beam (FIB) for the TEM foils, respectively.

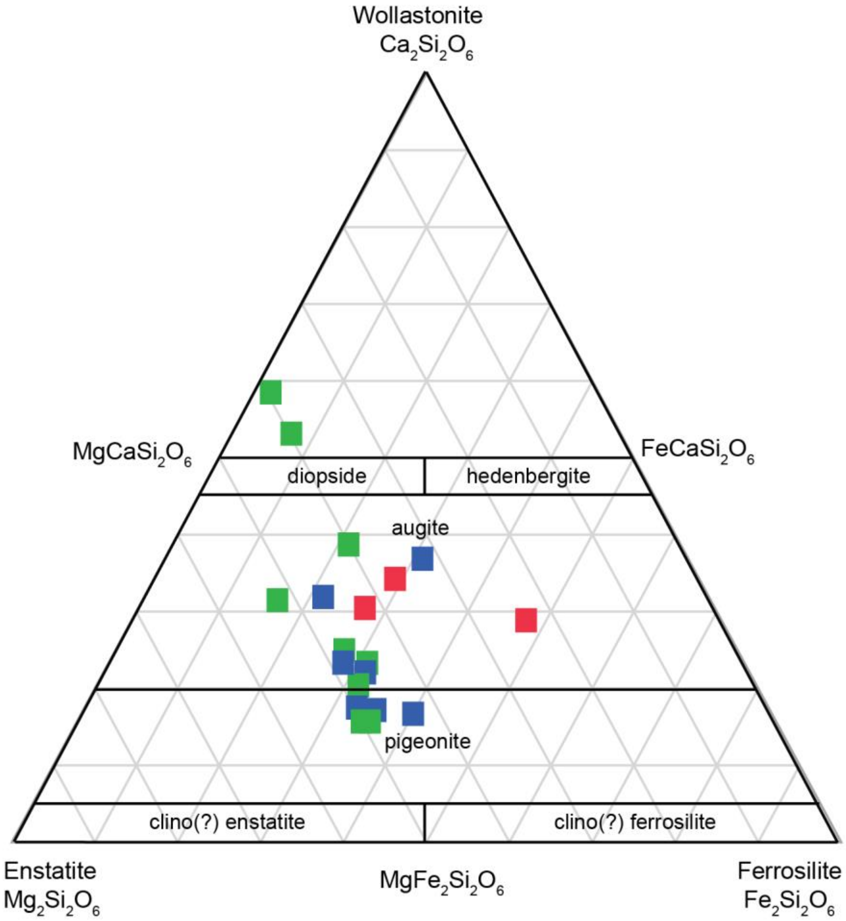

Fig. S5. Clinopyroxene ternary diagram of $Ca_2Si_2O_6$, $Mg_2Si_2O_6$, and $Fe_2Si_2O_6$.

The FEG-EPMA clinopyroxene point analyses from clinopyroxene-magnetite globules in the immiscible melt inclusions indicate a bimodality in the data; analyses plot primarily in the augite and pigeonite fields. Two analyses are highly depleted in Fe and Mg, and plot outside of clinopyroxene field. Color coding: red squares – clinopyroxene analyses from LCO-1 sample, blue squares – LAC-AND sample, and blue squares – LCO-9 sample.

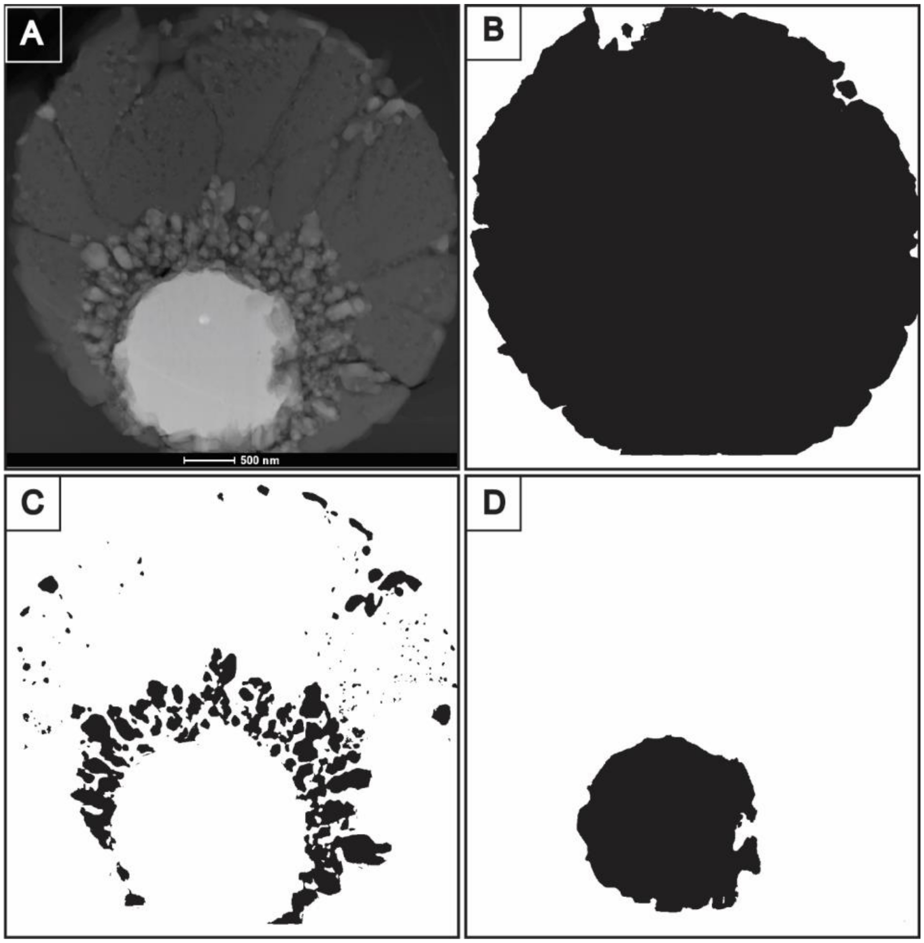

Fig. S6. Area masks used in calculation of modal percent of different phases.

(**A**) TEM image of globules with Cu-sulfide used in the creation of binary images in ImageJ 1.52a software. (**B**) Binary image of the whole globules area. (**C**) Binary image of magnetite crystals area. (**D**) Binary image of the Cu-sulfide area. Figure associated with the ‘Modal percentage and volume calculations of different phases in melt inclusions’ section in Methods section. Sample LCO-1_#4796 (Table S9).

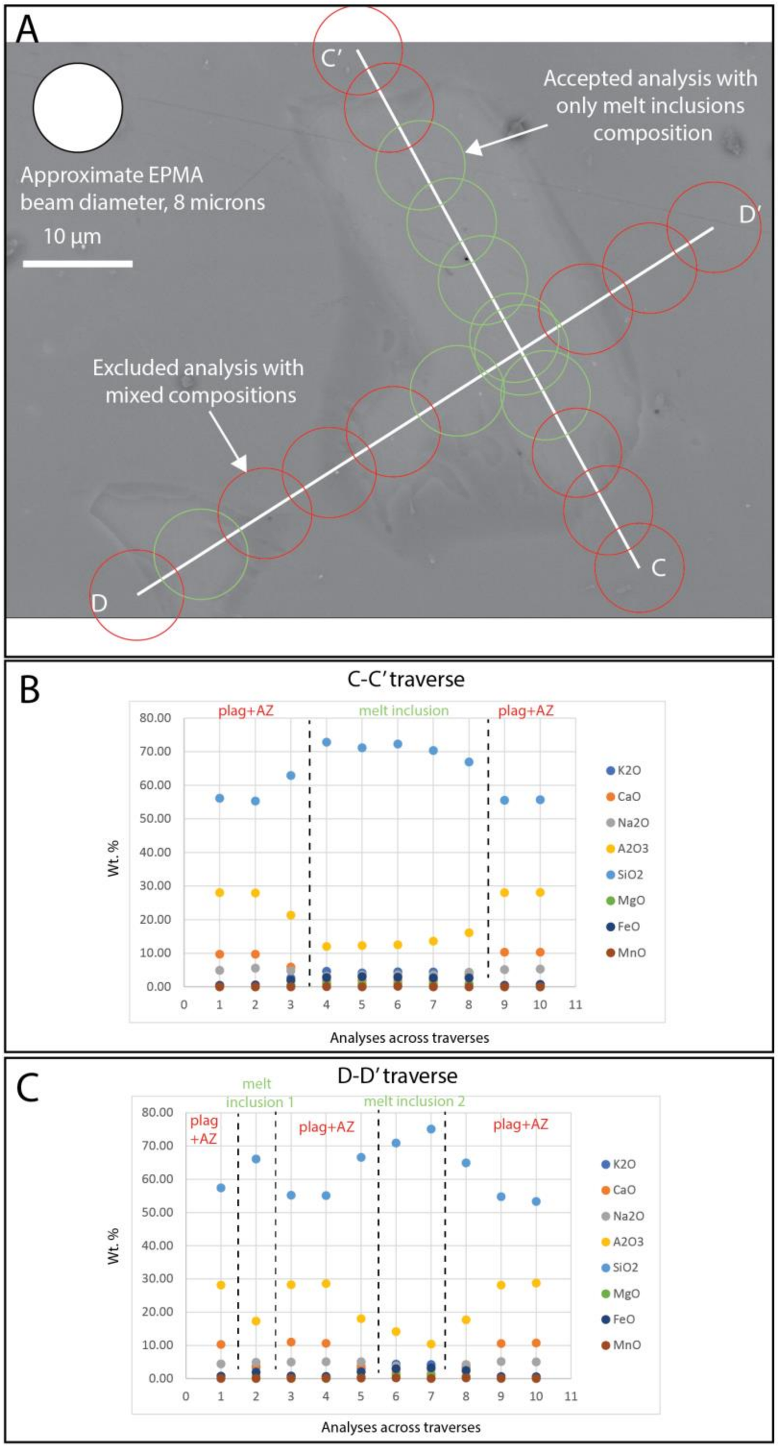

Fig. S7. Composition of homogenized melt inclusions obtained by EPMA traverses (example based on sample LCO-1).
(**A**) BSE images showing the EPMA traverses 'C-C' and D-D' crossing two melt inclusions. Only analyses marked as green circles were included into the average composition of homogenized melt inclusions (Table S1 and S11). (**B-C**) The geochemical composition of analyses of C-C' and D-D' traverses showing the recognized analyses of melt inclusions, albitized zones (AZ), and host plagioclase (plag).

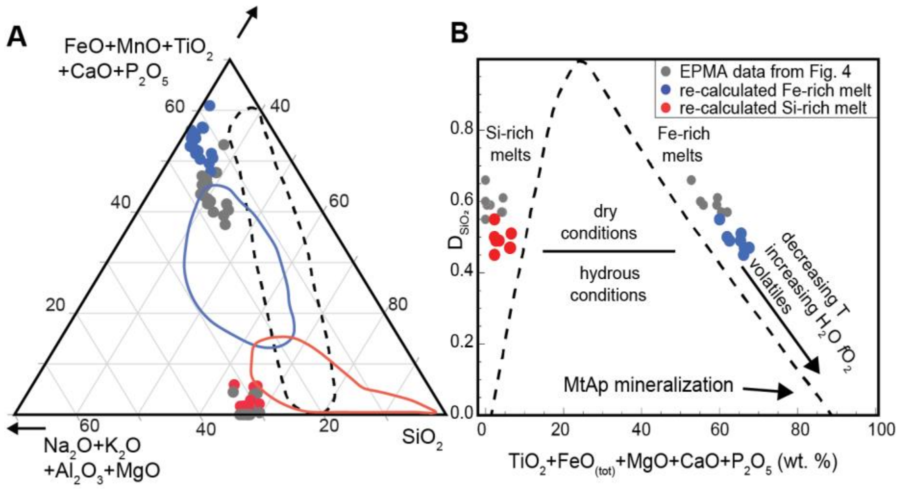

Fig. S8. Figure 5 plots with re-calculated composition of Fe-rich and Si-rich melts.
(**A**) Na_2O + K_2O + Al_2O_3 + MgO vs FeO_{tot} + MnO + TiO_2 + CaO + P_2O_5 vs SiO_2 ternary plot showing the immiscibility gap between Fe-rich and Si-rich melts (after *34*) based on re-calculated composition of FEG-EPMA point analyses. (**B**) Plot of SiO_2 partitioning between conjugate melts as a function of elements entering the Fe-rich melt (TiO_2 + FeO_{tot} + MgO + CaO + P_2O_5) with plotted re-calculated average compositions of conjugate melts in individual melt inclusions from this study (after [3,4]).

The data used to represent Fe-rich and Si-rich melts in Fig. 5 a-b includes FEG-EPMA point analyses of cpx from the cpx-mt globules and the high-SiO_2 dacite glass enclosed in the melt inclusions, respectively. The cpx composition from cpx-mt globules does not fully reflect the composition of cpx-mt globules, thus Figure S10 also includes recalculated FEG-EPMA point analyses. The re-calculated composition of Fe-rich melt is based on calculated phase-proportions: 82% of cpx FEG-EPMA point analyses (Table S3) and 18 % of stoichiometric mt crystals within cpx-mt globules (Table S6). Similarly, high-SiO_2 dacite glass host ~ 5 modal % of cpx crystals, that were included in the Si-rich melt compositions by re-calculating 95% of high-SiO_2 dacite glass FEG-EPMA point analyses (Table S2) and 5 % of stoichiometric cpx. The re-calculated compositions of the Fe-rich and Si-rich melt do not vary significantly from the EPMA point analyses plotted on Figure 5a-b and does not change the main results.

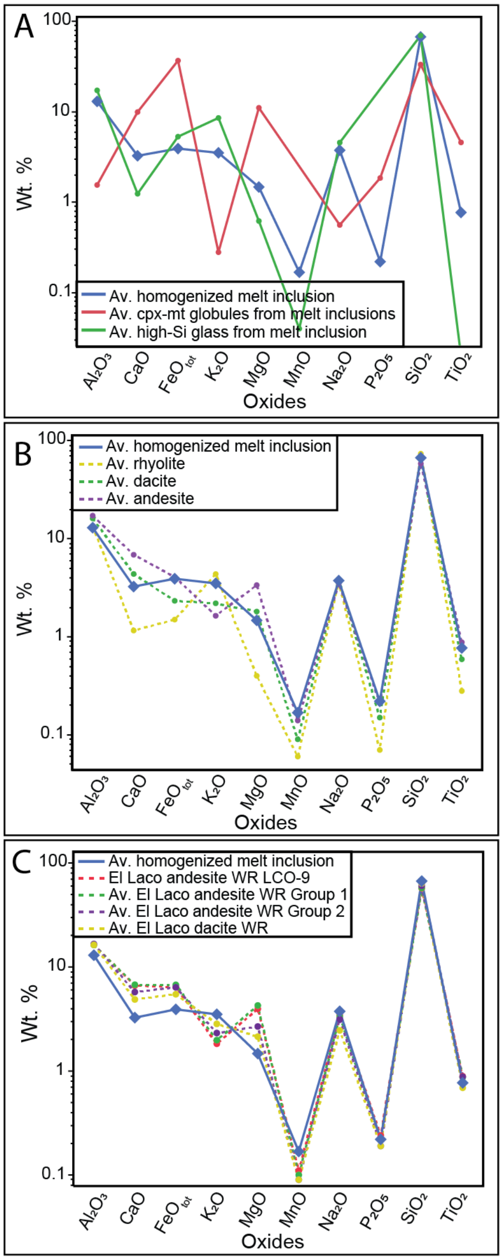

Fig. S9. Figure 6 with log scale on y-axis.

The average compositions of whole rock (WR) analyses of group 1 andesite (n=7), group 2 andesite (n=9), and dacite (n=2) from El Laco is from [5]. The two groups of andesite are based on variations in the bulk rock Sm-Nd and Rb-Sr isotopic compositions as shown in Figure 2 of [5].

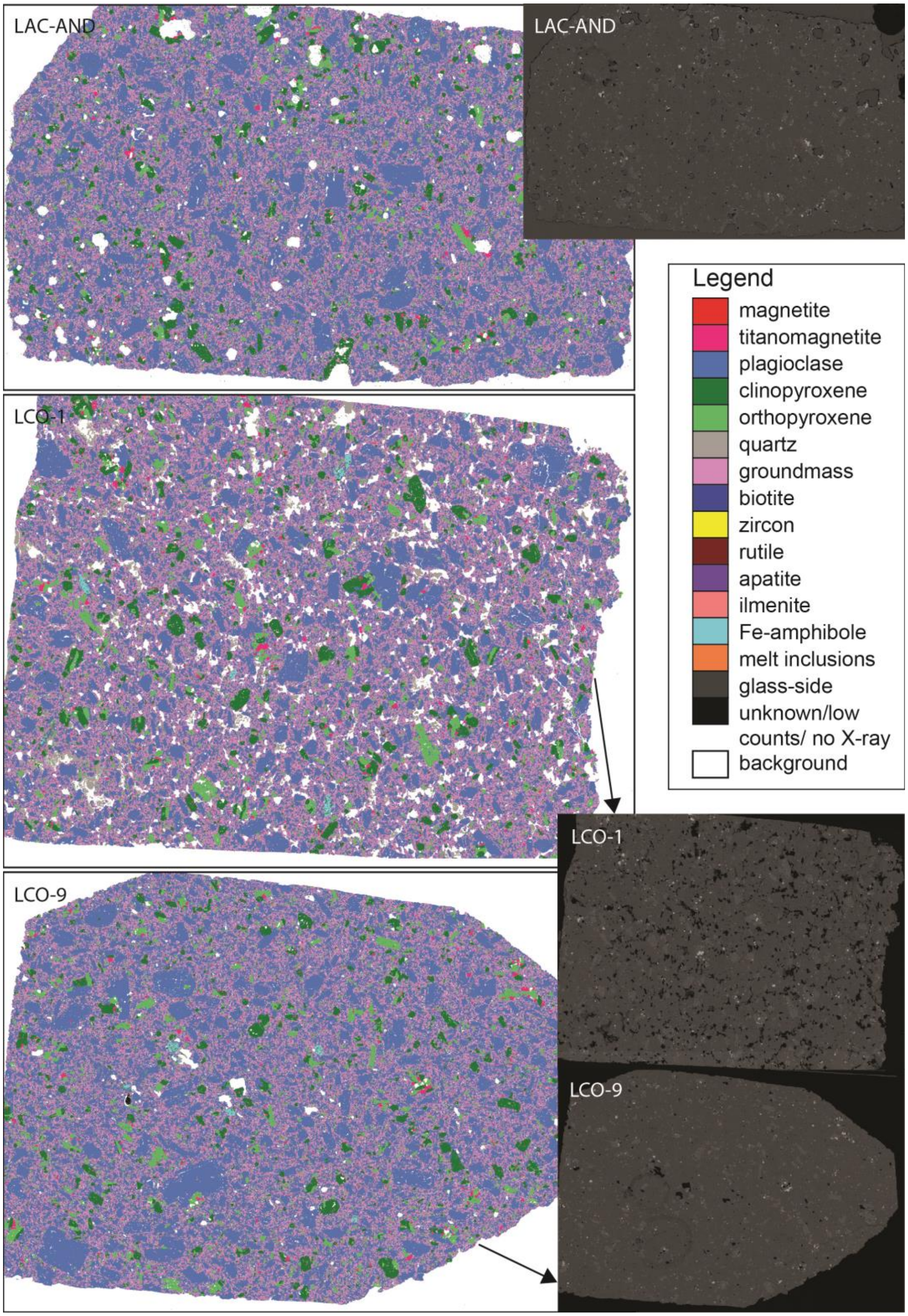

Fig. S10. Results of mineral liberation analysis (MLA) modified from Pietruszka, D. K., Hanchar, J. M., Tornos, F., Whitehouse, M. J., & Velasco, F. Tracking isotopic sources of immiscible melts at the enigmatic magnetite-(apatite) deposit at El Laco, Chile, using Pb isotopes. Geol. Soc. Am. Bull. 1–18 (2023). DOI:10.1130/B36506.1[6] in accordance with GSA's fair use policy.

Table. S1. Average compositions of crystallized melts in melt inclusions and homogenized melt inclusions.

Oxide and elements	Av. cpx composition from cpx-mt globules [wt.%] by FEG-EPMA	SD	Av. high-SiO_2 dacite glass [wt.%] by FEG-EPMA	SD	Re-calculated composition of Fe-rich melt (82% of cpx and 18 % of stoichiometric mt)	Re-calculated composition of Si-rich melt (95% of cpx and 5 % of stoichiometric mt)	Av. composition of homogenized melt inclusions	SD
SiO_2 wt%	40.48	2.46	67.94	1.40	33.19	67.11	67.21	5.02
TiO_2 wt%	5.59	2.18	<LOD	NA	4.58	0.02	0.77	0.62
Al_2O_3 wt%	1.89	0.53	18.05	0.76	1.55	17.22	13.01	3.20
FeO (tot) wt%	24.35	4.39	4.32	0.22	36.72	5.30	3.92	1.73
MnO wt%	-	-	-	-	-	0.04	0.17	0.07
MgO wt%	13.48	2.33	<LOD	NA	11.05	0.62	1.47	1.19
CaO wt%	12.10	4.51	0.80	0.60	9.92	1.24	3.26	1.74
Na_2O wt%	0.68	0.35	4.80	1.26	0.56	4.56	3.75	0.96
K_2O wt%	0.34	0.11	8.99	2.63	0.28	8.54	3.51	0.87
P_2O_5 wt%	2.26	1.29	<LOD	NA	1.85	0.00	0.22	0.26
Cl wt%	0.26	0.04	0.13	NA	0.21	0.12	0.08	0.07
F (ppm)	-	-	-	-	-		270.00	42.43
Cu (ppm)	-	-	-	-	-		162.06	73.07
S (ppm)	-	-	-	-	-		117.27	122.85
Total	100.84		101.63		99.93	104.78	97.51	

Notes: Re-calculated composition of Fe-rich and Si-rich melt based on 19 and 11 FEG-EPMA analyses, respectively, of clinopyroxene in cpx-mt globules and high-SiO_2 dacite glass from eight immiscible melt inclusions and calculation of phase proportions (Table S2-S3, S6). The average composition of homogenized melt inclusions is based on seventy analyses of nineteen homogenized melt inclusions (Table S11). Abbreviations: SD – standard deviation, NA – not applicable.

Table S2. Results of FEG-EPMA spot analyses of the high-SiO_2 dacite glass in melt inclusions

Sample ID	melt inclusion ID	Na_2O %	Na Elemental %	Na Detection Limits	Na % Errors	MgO %	Mg Elemental %	Mg Detection Limits	Mg % Errors	Al_2O_3 %	Al Elemental %	Al Detection Limits	Al % Errors	SiO_2 %	Si Elemental %	Si Detection Limits	Si % Errors
Un 10 LAC-AND_plag#10_reg#4-rhy-4	1	6.85	5.08	0.12	5.68	<LOD	<LOD	0.06	100.00	18.39	9.73	0.08	2.76	67.98	31.78	0.08	1.41
Un 11 LAC-AND_plag#10_reg#4-rhy-5	1	4.90	3.63	0.07	6.67	<LOD	<LOD	0.07	100.00	19.38	10.26	0.08	2.69	69.50	32.49	0.09	1.40
Un 21 LAC-AND_plag#10_reg#1-2-rhy-1	2	4.61	3.42	0.12	6.99	<LOD	<LOD	0.07	100.00	18.03	9.54	0.09	2.79	68.62	32.08	0.08	1.40
Un 22 LAC-AND_plag#10_reg#1-2-rhy-2	2	7.64	5.67	0.09	5.33	<LOD	<LOD	0.07	100.00	18.87	9.99	0.09	2.74	67.84	31.71	0.09	1.42
Un 25 LAC-AND_plag#10_reg#1-2-rhy-5	3	3.73	2.77	0.13	7.88	<LOD	<LOD	0.06	100.00	17.05	9.02	0.08	2.86	69.69	32.58	0.09	1.39
Un 54 LCO-9_plag#4_reg#4-rhy-1	12	4.03	2.99	0.14	7.61	<LOD	<LOD	0.05	100.00	17.80	9.42	0.06	2.79	67.50	31.55	0.08	1.42
Un 55 LCO-9_plag#4_reg#4-rhy-2	12	4.46	3.31	0.07	6.97	<LOD	<LOD	0.04	100.00	17.94	9.50	0.08	2.79	67.93	31.75	0.09	1.41
Un 57 LCO-9_plag#2_reg#5-rhy-1	13	4.23	3.14	0.15	7.47	<LOD	<LOD	0.06	100.00	17.49	9.26	0.10	2.84	66.89	31.27	0.08	1.42
Un 59 LCO-9_plag#2_reg#5-rhy-2	14	4.22	3.13	0.13	7.38	<LOD	<LOD	0.07	100.00	18.87	9.99	0.08	2.73	68.70	32.11	0.08	1.40
Un 62 LCO-9_plag#3_reg#1-rhy-1	15	4.03	2.99	0.14	7.60	<LOD	<LOD	0.06	100.00	17.06	9.03	0.08	2.87	68.18	31.87	0.08	1.41
Un 67 LCO-9_plag#3_reg#4-rhy-2	16	4.08	3.03	0.08	7.37	<LOD	<LOD	0.07	100.00	17.64	9.34	0.09	2.82	64.54	30.17	0.07	1.44
**Average oxide %**		4.80				**<LOD**				**18.05**				**67.94**			
**SD**		1.26				NA				0.76				1.40			

Notes: any analyses that are less than the minimum detection limit (LOD) are listed as <LOD, and the respective errors for those analyses are expressed at 100%; NA – not applicable

Table S2. Cont.

Sample ID	melt inclusion ID	K_2O %	K Elemental %	K Detection Limits	K % Errors	CaO %	Ca Elemental %	Ca Detection Limits	Ca % Errors	TiO_2 %	Ti Elemental %	Ti Detection Limits	Ti % Errors	P_2O_5 %	P Elemental %	P Detection Limits	P % Errors
Un 10 LAC-AND_plag#10_reg#4-rhy-4	1	8.25	6.85	0.20	4.83	<LOD	<LOD	0.26	100.00	<LOD	<LOD	0.25	100.00	<LOD	<LOD	0.21	100.00
Un 11 LAC-AND_plag#10_reg#4-rhy-5	1	5.19	4.31	0.19	6.17	<LOD	<LOD	0.22	100.00	<LOD	<LOD	0.25	100.00	<LOD	<LOD	0.20	100.00
Un 21 LAC-AND_plag#10_reg#1-2-rhy-1	2	10.64	8.83	0.16	4.16	<LOD	<LOD	0.21	100.00	<LOD	<LOD	0.26	100.00	<LOD	<LOD	0.18	100.00
Un 22 LAC-AND_plag#10_reg#1-2-rhy-2	2	4.71	3.91	0.23	6.71	1.22	0.87	0.24	18.67	<LOD	<LOD	0.24	100.00	<LOD	<LOD	0.20	100.00
Un 25 LAC-AND_plag#10_reg#1-2-rhy-5	3	11.71	9.72	0.19	4.00	<LOD	<LOD	0.25	100.00	<LOD	<LOD	0.30	100.00	<LOD	<LOD	0.23	100.00
Un 54 LCO-9_plag#4_reg#4-rhy-1	12	7.33	6.08	0.21	5.19	0.37	0.27	0.21	44.41	<LOD	<LOD	0.25	100.00	<LOD	<LOD	0.18	100.00
Un 55 LCO-9_plag#4_reg#4-rhy-2	12	11.77	9.77	0.20	4.00	<LOD	<LOD	0.29	100.00	<LOD	<LOD	0.24	100.00	<LOD	<LOD	0.23	100.00
Un 57 LCO-9_plag#2_reg#5-rhy-1	13	9.88	8.20	0.17	4.34	<LOD	<LOD	0.22	100.00	<LOD	<LOD	0.24	100.00	<LOD	<LOD	0.17	100.00
Un 59 LCO-9_plag#2_reg#5-rhy-2	14	6.89	5.72	0.22	5.38	<LOD	<LOD	0.24	100.00	<LOD	<LOD	0.25	100.00	<LOD	<LOD	0.20	100.00
Un 62 LCO-9_plag#3_reg#1-rhy-1	15	11.29	9.37	0.22	4.12	<LOD	<LOD	0.27	100.00	<LOD	<LOD	0.26	100.00	<LOD	<LOD	0.21	100.00
Un 67 LCO-9_plag#3_reg#4-rhy-2	16	11.23	9.32	0.20	4.09	<LOD	<LOD	0.25	100.00	<LOD	<LOD	0.26	100.00	<LOD	<LOD	0.17	100.00
**Average oxide %**		**8.99**				**0.80**				**<LOD**				**<LOD**			
**SD**		2.63				0.60				NA				NA			

Notes: any analyses that are less than the minimum detection limit (LOD) are listed as <LOD, and the respective errors for those analyses are expressed at 100%; NA – not applicable

Table S2. Cont.

Sample ID	melt inclusion ID	Cl %	Cl Detection Limits	Cl % Errors	FeO %	Fe Elemental %	Fe Detection Limits	Fe % Errors	Oxide Totals
Un 10 LAC-AND_plag#10_reg#4-rhy-4	1	0.13	0.13	56.57	<LOD	<LOD	2.34	100.00	102.62
Un 11 LAC-AND_plag#10_reg#4-rhy-5	1	<LOD	0.17	100.00	4.34	3.37	2.17	32.02	103.18
Un 21 LAC-AND_plag#10_reg#1-2-rhy-1	2	<LOD	0.20	0.00	<LOD	<LOD	2.41	100.00	103.76
Un 22 LAC-AND_plag#10_reg#1-2-rhy-2	2	<LOD	0.17	100.00	<LOD	<LOD	2.51	100.00	101.57
Un 25 LAC-AND_plag#10_reg#1-2-rhy-5	3	<LOD	0.14	100.00	<LOD	<LOD	2.48	100.00	99.75
Un 54 LCO-9_plag#4_reg#4-rhy-1	12	<LOD	0.17	100.00	<LOD	<LOD	2.41	100.00	98.11
Un 55 LCO-9_plag#4_reg#4-rhy-2	12	<LOD	0.17	100.00	<LOD	<LOD	2.53	100.00	102.16
Un 57 LCO-9_plag#2_reg#5-rhy-1	13	<LOD	0.15	100.00	<LOD	<LOD	2.33	100.00	101.34
Un 59 LCO-9_plag#2_reg#5-rhy-2	14	<LOD	0.15	100.00	4.09	3.18	2.38	36.94	103.03
Un 62 LCO-9_plag#3_reg#1-rhy-1	15	<LOD	0.15	100.00	<LOD	<LOD	2.46	100.00	100.01
Un 67 LCO-9_plag#3_reg#4-rhy-2	16	<LOD	0.17	100.00	4.54	3.53	2.30	32.38	102.36
**Average oxide %**		**0.13**			**4.32**				**101.63**
**SD**		NA			0.22				

Notes: any analyses that are less than the minimum detection limit (LOD) are listed as <LOD, and the respective errors for those analyses are expressed at 100%; NA – not applicable

Table S3. Results of FEG-EPMA spot analyses of the clinopyroxene from cpx-mt globules hosted by melt inclusions.

Sample ID	melt inclusion ID	Na_2O %	Na Elemental %	Na Detection Limits	Na % Errors	MgO %	Mg Elemental %	Mg Detection Limits	Mg % Errors	Al_2O_3 %	Al Elemental %	Al Detection Limits	Al % Errors	SiO_2 %	Si Elemental %	Si Detection Limits	Si % Errors
Un 6 LAC-AND_plag#10_reg#4-cpx-5	1	0.30	0.23	0.12	37.74	14.10	8.51	0.07	2.35	1.47	0.78	0.10	11.43	39.09	18.27	0.09	1.85
Un 12 LAC-AND_plag#10_reg#1-2-cpx-1	2	0.51	0.38	0.14	27.26	11.36	6.85	0.07	2.61	1.67	0.89	0.09	10.34	39.47	18.45	0.10	1.84
Un 15 LAC-AND_plag#10_reg#1-2-cpx-4	2	1.00	0.74	0.16	18.10	13.97	8.43	0.06	2.35	1.50	0.80	0.07	10.65	39.65	18.54	0.11	1.84
Un 17 LAC-AND_plag#10_reg#1-2-cpx-6	2	0.72	0.53	0.13	21.33	15.51	9.35	0.06	2.23	1.75	0.92	0.09	10.13	40.68	19.02	0.06	1.81
Un 18 LAC-AND_plag#10_reg#1-2-cpx-7	3	0.60	0.44	0.17	26.36	14.66	8.84	0.06	2.29	1.48	0.78	0.10	11.59	37.99	17.76	0.09	1.88
Un 19 LAC-AND_plag#10_reg#1-2-cpx-8	4	0.45	0.33	0.15	30.94	15.40	9.28	0.07	2.24	1.83	0.97	0.06	9.34	41.12	19.22	0.09	1.81
Un 20 LAC-AND_plag#10_reg#1-2-cpx-9	4	0.98	0.73	0.11	16.86	14.18	8.55	0.07	2.34	1.74	0.92	0.10	10.47	38.92	18.19	0.07	1.85
Un 27 LCO-1_plag#5_reg#1-cpx-1	5	0.39	0.29	0.16	35.86	7.36	4.44	0.07	3.29	2.00	1.06	0.10	9.52	38.62	18.05	0.09	1.86
Un 34 LCO-1_plag#5_reg#2-cpx-3	6	0.81	0.60	0.13	19.68	10.82	6.52	0.06	2.68	1.31	0.69	0.09	11.90	38.38	17.94	0.09	1.86
Un 44 LCO-1_plag#5_reg#4-cpx-2	9	0.38	0.28	0.14	33.74	13.81	8.33	0.08	2.37	1.73	0.91	0.10	10.40	36.60	17.11	0.09	1.91
Un 47 LCO-9_plag#4_reg#2-cpx-1	10	1.76	1.31	0.13	12.23	10.08	6.08	0.07	2.80	3.05	1.61	0.08	7.19	43.82	20.48	0.09	1.75
Un 48 LCO-9_plag#4_reg#2-cpx-2	11	0.84	0.62	0.13	19.22	15.68	9.46	0.06	2.21	0.92	0.49	0.10	15.74	46.40	21.69	0.08	1.70
Un 49 LCO-9_plag#4_reg#2-cpx-3	11	0.89	0.66	0.09	16.99	10.89	6.57	0.07	2.67	2.96	1.57	0.08	7.34	43.40	20.29	0.09	1.76
Un 53 LCO-9_plag#4_reg#4-cpx-2	12	0.95	0.71	0.13	17.79	14.28	8.61	0.07	2.33	1.75	0.93	0.10	10.24	40.28	18.83	0.08	1.82
Un 56 LCO-9_plag#2_reg#5-cpx-1	13	0.56	0.42	0.17	27.68	13.38	8.07	0.06	2.39	2.55	1.35	0.07	7.79	44.42	20.76	0.09	1.74
Un 58 LCO-9_plag#2_reg#5-cpx-2	14	0.25	0.19	0.13	44.87	16.12	9.72	0.08	2.20	2.04	1.08	0.09	9.16	39.22	18.33	0.10	1.85
Un 60 LCO-9_plag#3_reg#1-cpx-1	15	0.65	0.48	0.11	21.56	14.13	8.52	0.08	2.35	2.10	1.11	0.09	9.13	41.15	19.24	0.08	1.80
Un 61 LCO-9_plag#3_reg#1-cpx-2	15	0.56	0.42	0.18	28.75	14.54	8.77	0.06	2.31	2.33	1.23	0.09	8.56	40.28	18.83	0.10	1.83
Un 65 LCO-9_plag#3_reg#4-cpx-2	16	0.37	0.27	0.13	33.97	15.86	9.56	0.07	2.22	1.70	0.90	0.10	10.52	39.61	18.52	0.10	1.84
**Average oxide %**		**0.68**				**13.48**				**1.89**				**40.48**			
**SD**		0.35				2.33				0.53				2.46			

Notes: any analyses that are less than the minimum detection limit (LOD) are listed as <LOD, and the respective errors for those analyses are expressed at 100%; NA – not applicable

Table S3. Cont.

Sample ID	melt inclusion ID	K_2O %	K Elemental %	K Detection Limits	K % Errors	CaO %	Ca Elemental %	Ca Detection Limits	Ca % Errors	TiO_2 %	Ti Elemental %	Ti Detection Limits	Ti % Errors	P_2O_5 %	P Elemental %	P Detection Limits	P % Errors
Un 6 LAC-AND_plag#10_reg#4-cpx-5	1	0.23	0.19	0.22	61.60	7.58	5.42	0.23	5.58	7.82	4.69	0.24	4.82	2.47	1.08	0.16	14.55
Un 12 LAC-AND_plag#10_reg#1-2-cpx-1	2	<LOD	<LOD	0.27	100.00	18.20	13.01	0.24	3.50	4.29	2.57	0.26	7.40	6.27	2.74	0.16	8.53
Un 15 LAC-AND_plag#10_reg#1-2-cpx-4	2	<LOD	<LOD	0.20	100.00	13.34	9.53	0.22	4.10	6.28	3.77	0.25	5.60	1.97	0.86	0.19	17.68
Un 17 LAC-AND_plag#10_reg#1-2-cpx-6	2	<LOD	<LOD	0.22	100.00	7.62	5.44	0.20	5.51	7.18	4.30	0.22	5.01	1.49	0.65	0.21	22.71
Un 18 LAC-AND_plag#10_reg#1-2-cpx-7	3	0.27	0.22	0.20	49.79	7.38	5.27	0.24	5.73	6.79	4.07	0.26	5.43	2.70	1.18	0.15	13.72
Un 19 LAC-AND_plag#10_reg#1-2-cpx-8	4	<LOD	<LOD	0.23	100.00	10.34	7.39	0.26	4.79	5.53	3.31	0.23	6.00	1.79	0.78	0.21	19.58
Un 20 LAC-AND_plag#10_reg#1-2-cpx-9	4	<LOD	<LOD	0.21	100.00	9.40	6.72	0.23	4.97	7.17	4.30	0.25	5.13	2.33	1.02	0.17	15.39
Un 27 LCO-1_plag#5_reg#1-cpx-1	5	<LOD	<LOD	0.23	100.00	12.56	8.97	0.28	4.30	7.72	4.63	0.27	5.04	1.68	0.74	0.22	20.80
Un 34 LCO-1_plag#5_reg#2-cpx-3	6	0.45	0.38	0.21	32.48	14.06	10.05	0.24	4.01	6.85	4.10	0.25	5.35	2.18	0.95	0.21	17.29
Un 44 LCO-1_plag#5_reg#4-cpx-2	9	<LOD	<LOD	0.21	100.00	13.87	9.91	0.24	4.05	6.96	4.17	0.25	5.28	3.86	1.68	0.20	11.71
Un 47 LCO-9_plag#4_reg#2-cpx-1	10	<LOD	<LOD	0.20	100.00	20.76	14.83	0.24	3.28	1.34	0.81	0.24	17.43	<LOD	<LOD	0.17	100.00
Un 48 LCO-9_plag#4_reg#2-cpx-2	11	<LOD	<LOD	0.25	100.00	13.15	9.40	0.25	4.20	1.17	0.70	0.24	19.56	0.33	0.14	0.13	55.90
Un 49 LCO-9_plag#4_reg#2-cpx-3	11	<LOD	<LOD	0.23	100.00	20.27	14.49	0.23	3.31	1.24	0.74	0.24	18.56	<LOD	<LOD	0.17	100.00
Un 53 LCO-9_plag#4_reg#4-cpx-2	12	<LOD	<LOD	0.25	100.00	8.45	6.04	0.29	5.47	7.32	4.39	0.24	5.05	1.03	0.45	0.26	34.18
Un 56 LCO-9_plag#2_reg#5-cpx-1	13	<LOD	<LOD	0.24	100.00	18.01	12.87	0.23	3.53	4.28	2.57	0.23	7.08	2.01	0.88	0.19	17.79
Un 58 LCO-9_plag#2_reg#5-cpx-2	14	<LOD	<LOD	0.22	100.00	7.19	5.14	0.22	5.77	6.91	4.15	0.27	5.44	2.58	1.12	0.24	16.28
Un 60 LCO-9_plag#3_reg#1-cpx-1	15	0.42	0.35	0.19	33.14	10.35	7.40	0.26	4.81	5.42	3.25	0.25	6.24	1.27	0.56	0.22	25.87
Un 61 LCO-9_plag#3_reg#1-cpx-2	15	<LOD	<LOD	0.22	100.00	10.38	7.42	0.27	4.81	5.31	3.18	0.27	6.52	2.54	1.11	0.16	14.40
Un 65 LCO-9_plag#3_reg#4-cpx-2	16	<LOD	<LOD	0.23	100.00	6.93	4.96	0.33	6.30	6.67	4.00	0.24	5.34	1.88	0.82	0.20	18.72
**Average oxide %**		**0.34**				**12.10**				**5.59**				**2.26**			
**SD**		0.11				4.51				2.18				1.29			

Notes: any analyses that are less than the minimum detection limit (LOD) are listed as <LOD, and the respective errors for those analyses are expressed at 100%; NA – not applicable

Table S3. Cont.

Sample ID	melt inclusion ID	Cl %	Cl Detection Limits	Cl % Errors	FeO %	Fe Elemental %	Fe Detection Limits	Fe % Errors	Oxide Totals
Un 6 LAC-AND_plag#10_reg#4-cpx-5	1	<LOD	0.20	100.00	30.00	23.32	2.43	6.20	103.13
Un 12 LAC-AND_plag#10_reg#1-2-cpx-1	2	<LOD	0.19	100.00	19.65	15.27	2.41	8.87	101.28
Un 15 LAC-AND_plag#10_reg#1-2-cpx-4	2	<LOD	0.16	100.00	25.25	19.63	2.26	6.85	103.17
Un 17 LAC-AND_plag#10_reg#1-2-cpx-6	2	<LOD	0.18	100.00	25.48	19.81	2.58	7.41	100.61
Un 18 LAC-AND_plag#10_reg#1-2-cpx-7	3	<LOD	0.18	100.00	28.71	22.32	2.53	6.59	100.58
Un 19 LAC-AND_plag#10_reg#1-2-cpx-8	4	<LOD	0.17	100.00	24.52	19.06	2.36	7.21	101.15
Un 20 LAC-AND_plag#10_reg#1-2-cpx-9	4	<LOD	0.17	100.00	26.66	20.72	2.41	6.81	101.47
Un 27 LCO-1_plag#5_reg#1-cpx-1	5	0.29	0.17	34.51	33.09	25.72	2.30	5.57	103.95
Un 34 LCO-1_plag#5_reg#2-cpx-3	6	0.29	0.16	33.40	24.28	18.87	2.56	7.71	99.43
Un 44 LCO-1_plag#5_reg#4-cpx-2	9	<LOD	0.17	100.00	22.02	17.11	2.57	8.40	99.57
Un 47 LCO-9_plag#4_reg#2-cpx-1	10	<LOD	0.19	100.00	17.62	13.69	2.61	10.34	98.51
Un 48 LCO-9_plag#4_reg#2-cpx-2	11	<LOD	0.17	100.00	23.65	18.38	2.45	7.62	102.06
Un 49 LCO-9_plag#4_reg#2-cpx-3	11	<LOD	0.14	100.00	19.81	15.40	2.56	9.18	99.79
Un 53 LCO-9_plag#4_reg#4-cpx-2	12	<LOD	0.19	100.00	24.77	19.26	2.53	7.48	99.11
Un 56 LCO-9_plag#2_reg#5-cpx-1	13	<LOD	0.17	100.00	15.08	11.72	2.48	11.42	100.43
Un 58 LCO-9_plag#2_reg#5-cpx-2	14	<LOD	0.18	100.00	27.68	21.52	2.43	6.64	102.09
Un 60 LCO-9_plag#3_reg#1-cpx-1	15	0.27	0.17	36.58	21.85	16.98	2.47	8.20	97.61
Un 61 LCO-9_plag#3_reg#1-cpx-2	15	0.20	0.16	45.92	24.42	18.98	2.46	7.44	100.69
Un 65 LCO-9_plag#3_reg#4-cpx-2	16	<LOD	0.19	100.00	28.15	21.88	2.48	6.62	101.25
**Average oxide %**		**0.26**			**24.35**				**100.84**
**SD**		0.04			4.39				

Notes: any analyses that are less than the minimum detection limit (LOD) are listed as <LOD, and the respective errors for those analyses are expressed at 100%; NA – not applicable

Table S4. Conditions of FEG-EPMA spot analyses on clinopyroxene and high-SiO_2 dacite glass in melt inclusions.

Element	X-Ray	Spectro	Crystal	On-Peak L	Order	Mode		On-Peak Cnts (s)	Hi-Peak Cnts (s)	Lo-Peak Cnts (s)	Standard
Na	ka	4	TAP	129.391	1	Diff	Linear	10	5	5	Talbite
Mg	ka	4	TAP	107.48	2	Diff	Linear	20	10	10	Diopside, USNM 117733
Al	ka	4	TAP	90.703	3	Diff	Linear	10	5	5	Corundum CT
Si	ka	4	TAP	77.601	4	Int	Linear	10	5	5	Rhyolitic Glass, USNM 72854 VG-568
K	ka	3	PET	119.935	1	Int	Linear	30	15	15	OR10 CT
Ca	ka	3	PET	107.669	2	Int	Linear	30	15	15	Wollastonite CT
Ti	ka	1	PET	88.213	1	Int	Linear	80	40	40	Rutile (CT)
P	ka	2	PET	197.122	1	Diff	Linear	40	20	20	Apatite CT
Cl	ka	2	PET	151.499	2	Int	Linear	30	15	15	Scapolite (Meionite), USNM R6600-1
Fe	Ll	5	LDE1	93.622	1	Diff	Polynomial	80	40	40	Fayalite, USNM 85276

Notes: Time Dependent Instensity (TDI) Correction: 5 sec interval for 1st element on each spectrometer;
Accelerating Voltage: 7KeV;
Beam Current: 1 nA;
Beam Diameter: Focused.

Table S5. Area analyses using ImageJ 1.52a and volume calculations of clinopyroxene-magnetite globules and high-SiO_2 dacite glass hosted by melt inclusions.

Sample name	Area [um²]			Si/Fe ratio	cpx-mt [%]	glass [%]	Volume [um³]			Si/Fe ratio	cpx-mt vol [%]	glass vol [%]
	Whole MI[1]	cpx-mt[2]	glass[3]				Whole MI; V_1	cpx-mt; V_2	glass; V_3			
LCO-1_plag7_reg4	286.32	47.52	238.80	5.0	16.6	83.4	3644.5	246.4	2776.0	11.3	6.8	76.2
LAC-AND_plag#10_reg#4	532.46	62.20	470.26	7.6	11.7	88.3	9242.6	369.0	7671.2	20.8	4.0	83.0
LCO-9_plag#3_reg#1	347.48	105.20	242.28	2.3	30.3	69.7	4872.5	811.7	2836.8	3.5	16.7	58.2
LCO-9_plag#3_reg#4	516.94	87.18	429.76	4.9	16.9	83.1	8841.5	612.3	6702.0	10.9	6.9	75.8
LCO-1_plag#5_reg#3	307.62	20.65	286.96	13.9	6.7	93.3	4058.6	70.6	3656.8	51.8	1.7	90.1
LCO-1_plag#5_reg#2	614.02	52.33	561.70	10.7	8.5	91.5	11445.7	284.7	10014.3	35.2	2.5	87.5
LCO-9_plag#2_reg#5_MI_A	289.81	48.75	241.05	4.9	16.8	83.2	3711.3	256.1	2815.3	11.0	6.9	75.9
LCO-9_plag#2_reg#5_MI_B	273.85	42.62	231.22	5.4	15.6	84.4	3409.0	209.3	2644.9	12.6	6.1	77.6
LAC-AND_plag#10_reg#1-2	1164.09	138.42	1025.68	7.4	11.9	88.1	29877.6	1225.0	24710.4	20.2	4.1	82.7
LCO-1_plag#5_reg#1	722.70	37.23	685.47	18.4	5.2	94.8	14615.0	170.9	13500.4	79.0	1.2	92.4
				Average	14.0	86.0						
				Median	13.7	86.3						
				Max	30.3	94.8						
				Min	5.2	69.7						
				SD	6.79	6.79						

Notes: [1]melt inclusion

[2]clinopyroxene-magnetite globules hosted by melt inclusions

[3] high-SiO_2 dacite glass hosted by melt inclusions

plag – plagioclase

reg - region

Table S6. Area analyses using ImageJ 1.52a of clinopyroxene and magnetite in clinopyroxene-magnetite globules.

No.	Sample name	Area [um²] Whole cpx-mt glob[1]	mt[2]	cpx[3]	cpx/mt ratio	mt [%]	cpx [%]
	LAC-AND_plag#10_reg#1-2						
1	Reg#1_cpx1	6.51	1.13	5.38	4.77	17.32	82.68
2	Reg#1_cpx2	15.17	3.88	11.29	2.91	25.58	74.42
3	Reg#1_cpx4	15.18	3.06	12.12	3.96	20.16	79.84
4	Reg#1_cpx5	6.32	1.17	5.15	4.42	18.45	81.55
5	Reg#1_cpx6	7.99	1.33	6.66	5.00	16.66	83.34
6	Reg#1_cpx7	14.46	2.87	11.59	4.04	19.85	80.15
	LAC-AND_plag#10_reg#4						
7	cpx-2	9.40	1.38	8.02	5.83	14.65	85.35
8	cpx-3	7.41	0.74	6.67	9.03	9.97	90.03
9	cpx-4	11.16	2.24	8.92	3.99	20.06	79.94
	LCO-9_plag#3_reg#1						
10	cpx-1	27.19	3.496	23.69	6.78	12.86	87.14
					Average	17.56	82.44
					Median	17.88	82.12
					Max	25.58	90.03
					Min	9.97	74.42
					SD	4.15	4.15

Notes: [1]whole clinopyroxene-magnetite globules
[2]magnetite crystals
[3]clinopyroxene
plag – plagioclase
reg - region

Table S7. Results of FEG-EPMA spot analyses of Cu-S mineral phase hosted by clinopyroxene-magnetite globules within melt inclusions.

Sample #	S Elemental Percents	Cu Elemental Percents	S Atomic Percents	Cu Atomic Percents	Elemental Totals	Atomic Totals	Calculated mineral phase
Un 11 LCO-1-Plag7-Reg4-CuS1-5kev-2	34.32	63.88	51.57	48.43	98.20	100.00	Covellite
Un 16 LCO-1-Plag8-Reg5-CuS1-5kev	34.50	68.73	49.87	50.13	103.23	100.00	Covellite
Un 19 LCO-1-Plag6-Reg6-CuS1-5kev-2	33.58	66.06	50.18	49.82	99.64	100.00	Covellite
Un 27 LCO-1-Plag8-Reg7-CuS1-5kev-1	32.11	68.14	48.29	51.71	100.25	100.00	Covellite
Un 41 LCO-1-Block-Plag3-Reg1-CuS-2	20.70	74.28	35.58	64.42	94.98	100.00	Digenite

Table S8. Calibration and reference standards for FEG-EPMA spot analyses of Cu-S mineral phase hosted by clinopyroxene-magnetite globules within melt inclusions.

Chalcopyrite (CT) (CuFeS)			**Calibration Standard[1]**	
Line	S	Cu	Fe	Total
197 G	35.64	35.72	30.43	101.79
198 G	35.09	32.65	30.43	98.16
199 G	34.93	33.95	30.43	99.30
200 G	34.95	35.50	30.43	100.87
201 G	33.64	34.04	30.43	98.11
202 G	34.71	35.44	30.43	100.58
203 G	36.05	34.16	30.43	100.65
204 B				
205 G	34.76	34.29	30.43	99.49
206 G	34.70	35.85	30.43	100.98

Bornite			**Reference Standard[2]**	
Line	S	Cu	Fe	Total
207 G	24.98	68.37	8.82	102.17
208 G	24.22	69.38	8.82	102.42
209 G	22.77	68.75	8.82	100.34
210 G	24.26	70.91	8.82	103.99
211 G	23.91	68.21	8.82	100.94
212 G	24.38	68.44	8.82	101.64
213 G	23.68	69.47	8.82	101.97
214 G	23.84	70.15	8.82	102.80
215 G	22.83	67.63	8.82	99.28
216 G	23.26	69.31	8.82	101.39

Notes: [1]Published: 34.94 S, 34.62 Cu, 30.43 Fe, 99.99 Total
[2]Published: 24.69 S, 66.48 Cu, 8.82 Fe, 99.99 Total

Table S9. Area analyses using ImageJ 1.52a and volume and Cu concentration calculations of Cu-S globules in clinopyroxene-magnetite globules hosted by melt inclusions.

	Area [um^2]						Volume [um^3]						% of Cu in Cu-S inclusion	
Sample name	Whole cpx-mt glob[1]	Cu-S	cpx[2] + mt[3]	cpx+mt/ Cu-S ratio	Cu-S [%]	cpx+mt [%]	Whole cpx-mt glob	Cu-S	cpx + mt	Cu-S/whole globule ratio	Cu-S vol [%]	cpx+mt vol [%]	digenite formula	covellite formula
LCO-1_plag#3_reg#1	7.88	2.08	5.80	2.78	26.43	73.57	16.63	2.26	10.50	13.59	13.6	63.1	10.61	9.03
LCO-1_plag#2_reg#1	5.87	0.63	5.23	8.27	10.79	89.21	10.69	0.38	9.01	3.54	3.5	84.3	2.77	2.36
LCO-1_plag#7_reg#4	8.87	0.79	8.09	10.29	8.86	91.14	19.88	0.52	17.30	2.64	2.6	87.0	2.06	1.75
LCO-1_plag#1_reg#1	9.41	0.70	8.71	12.46	7.43	92.57	21.72	0.44	19.35	2.02	2.0	89.1	1.58	1.35
LCO-1_plag#1_reg#2	6.97	0.26	6.70	25.39	3.79	96.21	13.83	0.10	13.05	0.74	0.7	94.4	0.58	0.49
LCO-1_plag#1_reg#3	2.11	0.16	1.95	12.21	7.57	92.43	2.31	0.05	2.05	2.08	2.1	88.9	1.63	1.38
LCO-1_plag#4_reg#4	3.22	0.30	2.92	9.81	9.25	90.75	4.35	0.12	3.76	2.81	2.8	86.4	2.20	1.87
LCO-1_plag#6_reg#6	11.56	1.97	9.58	4.86	17.05	82.95	29.51	2.08	22.30	7.04	7.0	75.5	5.50	4.68
LCO-1-Plag8_Reg5-CuS1	5.49	0.70	4.79	6.87	12.71	87.29	9.68	0.44	7.89	4.53	4.5	81.5	3.54	3.01
LCO-1_#4796	15.82	2.46	13.35	5.42	15.58	84.42	47.32	2.91	36.70	6.15	6.1	77.6	4.80	4.09
				Average	11.95	88.05						Average	3.53	3.00
				Median	10.02	89.98						Median	2.48	2.11
				Max	26.43	96.21						Max	10.61	9.03
				Min	3.79	73.57						Min	0.58	0.49
				SD	6.11	6.11								

Notes: [1]whole clinopyroxene-magnetite globules; [2]clinopyroxene; [3]magnetite; plag – plagioclase; reg – region

Table S10. Area analyses using ImageJ 1.52a of nano-melt inclusions with ilmenite crystals hosted by apatite in clinopyroxene-magnetite globules in melt inclusions.

Area calculations [nm]				area [%]
Phase	Area [nm]	Phase	Area [nm]	% of apatite area within globules
Whole Globules	34979000			4.83
Apatite	1690000			% of nMI's area in apatite
nMI[1] in apatite total	226002			13.37
nMI in apatite (# of globules area analysis):		Ilmenite[2] crystals in given nMI in apatite (# of area analysis):		% of ilmenite area in given nMI
1	3275	1	289	8.82
3	3281	3	192	5.86
5	21649	4	1935	8.94
6	14352	5	1887	13.15
8	4918	6	516	10.49
9	5354	9	486	9.07
12	5086	10	920	18.08
14	6058	11	1033	17.05
15	18199	13	1925	10.58
16	11247	15	2317	20.60

Notes: [1]nano melt inclusions

Table. S11. Results of EPMA traverse analyses of the eighteen homogenized melt inclusions.

Sample ID	melt inclusion ID	K_2O wt. %	K SD (%)	K DL (ppm^{-1s})	CaO wt. %	Ca SD (%)	Ca DL (ppm^{-1s})	Na_2O wt. %	Na SD (%)	Na DL (ppm^{-1s})	Al_2O_3 wt. %	Al SD (%)	Al DL (ppm^{-1s})	SiO_2 wt. %	Si SD (%)	Si DL (ppm^{-1s})	MgO wt. %	Mg SD (%)	Mg DL (ppm^{-1s})
LAC-and 2C Line 002	1	4.46	2.32	103.73	1.31	4.44	93.96	2.58	6.94	223.55	9.37	2.38	308.67	73.14	0.82	414.81	0.37	16.91	214.75
LAC-and 2C Line 003		4.59	2.27	87.94	1.98	3.67	127.46	2.85	6.74	303.64	12.44	2.05	273.90	68.39	0.86	406.60	0.50	13.62	193.86
LAC-and 2D Line 003		3.30	2.72	110.53	3.74	2.56	112.34	4.00	5.56	296.86	16.65	1.77	316.78	64.37	0.89	402.59	0.24	25.67	258.08
LAC-and 2D Line 004		4.39	2.33	102.92	2.18	3.47	128.52	1.84	8.35	209.91	9.59	2.36	303.35	69.57	0.85	419.88	0.62	13.01	268.50
**AVERAGE**		**4.19**			**2.30**			**2.82**			**12.01**			**68.87**			**0.43**		
**1σ SD**		**0.60**			**1.03**			**0.90**			**3.39**			**3.61**			**0.16**		
LAC-and 3E Line 003	2	4.02	2.44	100.44	2.06	3.56	119.80	3.68	5.75	261.93	12.37	2.07	355.18	67.93	0.86	412.44	0.18	26.49	181.65
LAC-and 3F Line 002		3.51	2.62	95.28	1.72	3.90	113.22	3.12	6.32	285.17	10.88	2.19	292.01	74.40	0.82	415.61	0.11	43.64	221.07
**AVERAGE**		**3.77**			**1.89**			**3.40**			**11.62**			**71.16**			**0.14**		
**1σ SD**		**0.36**			**0.24**			**0.40**			**1.05**			**4.57**			**0.05**		
LAC-and 5J Line 003	3	3.26	2.72	98.46	2.98	2.87	103.29	2.85	6.73	289.82	12.55	2.08	326.70	63.43	0.90	437.16	3.95	4.26	207.53
LAC-and 5K Line 002		3.09	2.80	102.92	2.75	3.00	108.57	3.40	5.98	209.91	12.26	2.11	305.54	63.62	0.90	434.92	4.27	4.14	258.08
LAC-and 5K Line 003		3.19	2.74	100.16	2.92	2.90	112.41	3.14	6.30	253.92	12.47	2.08	306.14	64.14	0.89	399.81	4.25	4.19	301.73
**AVERAGE**		**3.18**			**2.88**			**3.13**			**12.43**			**63.73**			**4.16**		
**1σ SD**		**0.09**			**0.12**			**0.28**			**0.15**			**0.37**			**0.18**		
LCO-1 p1_t1_A Line 003	4	4.32	2.35	100.99	2.76	3.01	107.90	3.71	5.80	279.54	13.19	1.99	248.56	68.83	0.86	386.71	0.80	10.36	208.30
LCO-1 p1_t1_A Line 004		4.39	2.35	113.78	2.57	3.18	129.91	4.03	5.45	188.19	13.85	1.94	241.92	66.92	0.87	380.89	0.67	11.97	234.51
LCO-1 p1_t1_A Line 006		3.29	2.71	100.16	4.28	2.40	123.94	4.80	5.09	328.10	18.01	1.70	234.06	63.77	0.90	360.06	0.58	13.06	235.13
LCO-1 p1_t1_A Line 007		4.18	2.40	99.88	2.72	3.05	117.44	4.17	5.39	236.41	14.67	1.88	226.19	68.19	0.87	387.51	0.86	9.85	201.57
LCO-1 p1_t1_A Line 008		4.24	2.37	97.89	2.86	2.96	110.39	4.58	5.16	271.23	15.00	1.86	222.69	67.32	0.87	406.66	0.81	10.36	214.75
LCO-1 p1_t1_A Line 009		4.29	2.36	96.16	2.60	3.12	114.87	4.16	5.53	342.97	14.75	1.87	202.87	67.35	0.87	367.12	0.77	10.83	233.21
LCO-1 p1_t1_B Line 002		4.53	2.31	104.54	1.67	3.96	108.98	2.59	6.94	223.55	10.55	2.22	244.37	74.72	0.82	428.55	0.76	10.79	219.69
**AVERAGE**		**4.18**			**2.78**			**4.00**			**14.29**			**68.16**			**0.75**		
**1σ SD**		**0.41**			**0.77**			**0.72**			**2.24**			**3.31**			**0.10**		

Notes: any analyses that are less than the minimum detection limit (LOD) are listed as <LOD, and the respective errors for those analyses are expressed at 100%.
SD – standard deviation; DL – detection limit

Table S11. Cont.

Sample ID	melt inclusion ID	FeO_{tot} wt. %	Fe SD (%)	Fe DL (ppm^{-1s})	MnO wt. %	Mn SD (%)	Mn DL (ppm^{-1s})	F wt. %	F ppm	F SD (%)	F DL (ppm^{-1s})	P_2O_5 wt. %	P ppm	P SD (%)	P DL (ppm^{-1s})	SO_3 wt. %	S ppm	S SD (%)	S DL (ppm^{-1s})
LAC-and 2C Line 002	1	2.97	6.60	386.49	0.10	77.62	341.09	<LOD	<LOD	100.00	90.31	0.02	69.83	14.66	8.36	<LOD	<LOD	100.00	8.02
LAC-and 2C Line 003		3.49	6.11	432.34	<LOD	100.00	310.66	0.03	300.00	43.33	82.66	0.19	837.93	1.87	8.47	0.03	120.14	7.22	7.90
LAC-and 2D Line 003		2.23	7.71	360.62	0.10	78.22	342.36	<LOD	<LOD	100.00	81.77	0.09	375.32	3.31	8.25	0.04	176.20	5.07	7.84
LAC-and 2D Line 004		4.68	5.11	386.49	<LOD	100.00	324.95	<LOD	<LOD	100.00	76.73	0.24	1038.68	1.60	8.26	0.08	300.34	3.28	8.06
**AVERAGE**		**3.34**			**0.10**			**0.03**	**300.00**			**0.13**	**580.44**			**0.05**	**198.89**		
**1σ SD**		**1.03**			**0.00**			**NA**	**NA**			**0.10**	**439.35**			**0.02**	**92.22**		
LAC-and 3E Line 003	2	1.80	9.30	459.71	<LOD	100.00	365.48	<LOD	<LOD	100.00	75.41	0.04	192.03	6.00	8.77	0.00	20.02	44.31	8.19
LAC-and 3F Line 002		1.49	10.23	423.36	<LOD	100.00	312.05	<LOD	<LOD	100.00	75.23	0.01	34.91	29.63	8.77	<LOD	<LOD	100.00	8.09
**AVERAGE**		**1.65**			**<LOD**			**<LOD**	**<LOD**			**0.03**	**113.47**			**0.00**	**20.02**		
**1σ SD**		**0.22**						**NA**	**NA**			**0.03**	**111.09**			**NA**	**NA**		
LAC-and 5J Line 003	3	4.53	5.29	452.23	0.17	51.10	354.72	<LOD	<LOD	100.00	77.40	0.09	397.14	3.17	8.36	<LOD	<LOD	100.00	8.00
LAC-and 5K Line 002		4.55	5.11	334.22	<LOD	100.00	391.68	<LOD	<LOD	100.00	87.78	0.21	929.58	1.75	8.59	<LOD	<LOD	100.00	8.02
LAC-and 5K Line 003		4.84	4.90	323.87	0.38	21.16	222.61	<LOD	<LOD	100.00	79.79	0.19	820.47	1.90	8.57	0.00	16.02	50.65	7.98
**AVERAGE**		**4.64**			**0.27**			**<LOD**	**<LOD**			**0.16**	**715.73**			**0.00**	**16.02**		
**1σ SD**		**0.18**			**0.15**			**NA**	**NA**			**0.06**	**281.25**			**NA**	**NA**		
LCO-1 p1_t1_A Line 003	4	2.91	6.68	385.85	<LOD	100.00	328.22	<LOD	<LOD	100.00	84.79	0.11	462.61	2.85	8.29	0.03	128.15	6.94	8.06
LCO-1 p1_t1_A Line 004		3.23	6.17	332.92	<LOD	100.00	400.36	<LOD	<LOD	100.00	84.14	0.22	947.03	1.72	8.30	0.05	200.23	4.58	7.91
LCO-1 p1_t1_A Line 006		2.63	7.02	361.05	<LOD	100.00	367.85	<LOD	<LOD	100.00	79.28	0.14	632.81	2.25	8.16	0.05	200.23	4.58	7.92
LCO-1 p1_t1_A Line 007		2.85	6.85	416.06	<LOD	100.00	380.52	<LOD	<LOD	100.00	78.77	0.23	1021.23	1.63	8.15	0.08	308.35	3.23	8.00
LCO-1 p1_t1_A Line 008		3.06	6.31	303.53	<LOD	100.00	389.46	<LOD	<LOD	100.00	78.99	0.13	576.08	2.40	8.10	0.05	220.25	4.24	7.96
LCO-1 p1_t1_A Line 009		2.65	7.27	452.10	0.14	53.94	299.38	<LOD	<LOD	100.00	79.29	0.13	584.80	2.40	8.31	0.05	220.25	4.26	7.94
LCO-1 p1_t1_B Line 002		3.24	6.31	393.48	0.11	63.16	308.61	<LOD	<LOD	100.00	81.46	0.13	580.44	2.41	8.28	0.05	180.21	5.13	8.04
**AVERAGE**		**2.94**			**0.13**			**<LOD**	**<LOD**			**0.16**	**686.43**			**0.05**	**208.24**		
**1σ SD**		**0.25**			**0.02**			**NA**	**NA**			**0.05**	**210.84**			**0.01**	**54.22**		

Table. S11. Cont.

Sample ID	melt inclusion ID	Cl wt. %	Cl ppm	Cl SD (%)	Cl DL (ppm^{-1s})	CuO wt. %	Cu ppm	Cu SD (%)	Cu DL (ppm^{-1s})	TiO_3 wt. %	Ti ppm	Ti SD (%)	Ti DL (ppm^{-1s})	Totals %
LAC-and 2C Line 002	1	0.00	20.00	63.04	6.20	<LOD	<LOD	100.00	23.35	0.04	257.72	9.01	16.11	93.93
LAC-and 2C Line 003		0.17	1700.00	1.38	6.30	0.01	55.92	80.62	23.52	1.43	8582.59	0.65	15.92	95.87
LAC-and 2D Line 003		0.14	1400.00	1.55	6.12	0.01	95.86	45.81	23.14	1.04	6257.14	0.78	15.48	95.26
LAC-and 2D Line 004		0.14	1440.00	1.54	6.29	0.05	383.46	11.66	23.09	1.39	8306.89	0.66	16.00	95.85
**AVERAGE**		**0.11**	**1140.00**			**0.02**	**178.41**			**0.98**	**5851.09**			
**1σ SD**		**0.08**	**758.42**			**0.02**	**178.69**			**0.65**	**3870.52**			
LAC-and 3E Line 003	2	0.01	110.00	12.96	6.36	<LOD	<LOD	100.00	23.36	0.12	701.23	3.67	16.14	95.65
LAC-and 3F Line 002		0.00	20.00	81.39	6.48	<LOD	<LOD	100.00	23.70	0.04	221.76	10.29	16.12	95.51
**AVERAGE**		**0.01**	**65.00**			**<LOD**	**<LOD**			**0.08**	**461.49**			
**1σ SD**		**0.01**	**63.64**			**NA**	**NA**			**0.06**	**339.04**			
LAC-and 5J Line 003	3	0.02	190.00	7.58	6.34	<LOD	<LOD	100.00	23.56	0.26	1582.27	1.92	15.94	94.73
LAC-and 5K Line 002		0.04	390.00	4.03	6.15	<LOD	<LOD	100.00	23.43	0.40	2397.37	1.44	16.09	94.95
LAC-and 5K Line 003		0.03	330.00	4.69	6.30	<LOD	<LOD	100.00	23.54	0.41	2457.31	1.42	16.29	94.98
**AVERAGE**		**0.03**	**303.33**			**<LOD**	**<LOD**			**0.36**	**2145.65**			
**1σ SD**		**0.01**	**102.63**			**NA**	**NA**			**0.08**	**488.82**			
LCO-1 p1_t1_A Line 003	4	0.14	1420.00	1.56	6.22	0.02	183.74	24.20	22.98	0.86	5160.34	0.88	15.85	98.31
LCO-1 p1_t1_A Line 004		0.16	1570.00	1.45	6.19	0.02	175.75	25.53	22.97	0.99	5903.53	0.81	15.81	98.58
LCO-1 p1_t1_A Line 006		0.16	1630.00	1.43	6.26	0.01	111.84	39.35	23.14	0.73	4351.23	0.97	15.85	98.20
LCO-1 p1_t1_A Line 007		0.19	1950.00	1.26	6.19	0.02	127.82	35.40	23.19	0.96	5759.69	0.82	15.75	98.58
LCO-1 p1_t1_A Line 008		0.19	1900.00	1.28	6.19	0.02	127.82	34.72	23.07	0.93	5543.92	0.84	15.69	98.41
LCO-1 p1_t1_A Line 009		0.19	1880.00	1.30	6.31	0.02	127.82	35.55	23.12	0.84	5028.49	0.89	15.64	98.33
LCO-1 p1_t1_B Line 002		0.18	1800.00	1.32	6.13	0.01	111.84	38.91	23.13	1.01	6047.37	0.80	16.08	98.47
**AVERAGE**		**0.17**	**1735.71**			**0.02**	**138.09**			**0.90**	**5399.22**			
**1σ SD**		**0.02**	**198.40**			**0.00**	**29.43**			**0.10**	**594.15**			

Table. S11. Cont.

**Sample ID**	**melt inclusion ID**	**K_2O wt. %**	**K SD (%)**	**K DL (ppm^{-1s})**	**CaO wt. %**	**Ca SD (%)**	**Ca DL (ppm^{-1s})**	**Na_2O wt. %**	**Na SD (%)**	**Na DL (ppm^{-1s})**	**Al_2O_3 wt. %**	**Al SD (%)**	**Al DL (ppm^{-1s})**	**SiO_2 wt. %**	**Si SD (%)**	**Si DL (ppm^{-1s})**	**MgO wt. %**	**Mg SD (%)**	**Mg DL (ppm^{-1s})**
LCO-1 p1_t2_C Line 004		4.61	2.27	92.30	1.78	3.83	110.96	3.21	6.22	255.79	12.01	2.10	297.59	72.82	0.83	401.82	1.02	8.92	208.99
LCO-1 p1_t2_C Line 005		4.16	2.40	99.88	1.86	3.72	107.07	3.53	6.01	316.05	12.27	2.07	269.30	71.18	0.84	408.52	0.82	10.48	235.13
LCO-1 p1_t2_C Line 006		4.45	2.33	104.27	1.77	3.92	132.46	3.58	5.82	221.41	12.50	2.04	251.67	72.25	0.84	447.61	0.89	9.53	192.29
LCO-1 p1_t2_C Line 007	5	4.39	2.34	105.07	2.00	3.59	110.55	3.60	5.88	283.50	13.62	1.96	260.41	70.32	0.85	424.37	0.83	10.13	213.32
LCO-1 p1_t2_C Line 008		4.02	2.44	99.60	2.85	2.98	120.47	4.37	5.21	193.03	16.08	1.80	282.42	66.93	0.88	415.56	0.88	10.08	240.92
LCO-1 p1_t2_D Line 006		4.35	2.34	86.01	1.79	3.83	111.36	3.78	5.81	332.91	14.11	1.92	230.67	70.88	0.85	419.03	0.99	9.18	215.49
LCO-1 p1_t2_D Line 007		4.22	2.38	93.80	1.42	4.43	126.75	2.86	6.60	238.29	10.39	2.24	227.00	75.09	0.82	440.02	1.01	9.15	233.21
**AVERAGE**		**4.31**			**1.92**			**3.56**			**13.00**			**71.35**			**0.92**		
**1σ SD**		**0.20**			**0.44**			**0.47**			**1.81**			**2.51**			**0.09**		
LCO-1 p1_t2_D Line 002	6	3.38	2.66	91.69	3.81	2.53	102.15	4.89	4.97	286.73	17.28	1.73	230.14	66.07	0.89	391.74	0.72	11.01	208.99
**AVERAGE**		**3.38**			**3.81**			**4.89**			**17.28**			**66.07**			**0.72**		
**1σ SD**		**NA**			**NA**			**NA**			**NA**			**NA**			**NA**		
LCO-1 p2_t3 E Line 004		2.43	3.18	100.72	5.57	2.08	123.73	3.30	6.23	260.10	9.64	2.38	248.31	66.78	0.88	440.36	4.64	3.99	252.68
LCO-1 p2_t3 E Line 005		2.47	3.14	92.90	4.09	2.45	119.12	2.86	6.77	281.92	9.90	2.35	251.18	67.99	0.87	437.47	5.22	3.73	240.92
LCO-1 p2_t3 E Line 006		2.89	2.88	89.52	2.42	3.23	112.25	2.57	7.36	325.27	9.39	2.39	205.49	68.86	0.86	426.67	2.73	5.18	178.27
LCO-1 p2_t3 E Line 007	7	3.19	2.75	92.90	2.24	3.40	123.73	2.76	6.71	197.59	9.55	2.34	192.16	71.61	0.84	398.63	1.72	6.69	207.53
LCO-1 p2_t3 F Line 005		3.03	2.82	92.60	2.31	3.33	118.43	2.86	6.80	299.10	8.71	2.48	237.62	69.66	0.85	415.61	1.81	6.45	184.98
LCO-1 p2_t3 F Line 006		3.08	2.81	101.82	2.81	2.97	108.91	2.24	7.64	250.39	10.80	2.22	241.92	70.74	0.85	367.12	2.74	5.16	193.04
LCO-1 p2_t3 F Line 007		2.17	3.41	107.97	2.54	3.15	113.22	2.96	6.49	234.38	9.34	2.38	234.58	74.96	0.82	425.86	1.78	6.59	227.22
**AVERAGE**		**2.75**			**3.14**			**2.79**			**9.62**			**70.08**			**2.95**		
**1σ SD**		**0.39**			**1.24**			**0.33**			**0.64**			**2.69**			**1.43**		
LCO-1 p1t4_G Line 008	8	2.12	3.39	92.30	8.52	1.67	123.14	4.57	5.12	197.59	21.20	1.59	382.31	57.51	0.96	405.01	0.73	11.41	238.99
LCO-1 p1t4_G Line 009		3.19	2.72	81.32	5.33	2.13	116.28	3.92	5.70	238.29	13.30	2.04	340.26	58.25	0.95	404.96	2.34	5.85	250.84
**AVERAGE**		**2.65**			**6.92**			**4.24**			**17.25**			**57.88**			**1.53**		
**1σ SD**		**0.76**			**2.25**			**0.46**			**5.59**			**0.52**			**1.14**		

Notes: any analyses that are less than the minimum detection limit (LOD) are listed as <LOD, and the respective errors for those analyses are expressed at 100%; SD – standard deviation; DL – detection limit

Table. S11. Cont.

Sample ID	melt inclusion ID	FeO_{tot} wt. %	Fe SD (%)	Fe DL (ppm^{-1s})	MnO wt. %	Mn SD (%)	Mn DL (ppm^{-1s})	F wt. %	F ppm	F SD (%)	F DL (ppm^{-1s})	P_2O_5 wt. %	P ppm	P SD (%)	P DL (ppm^{-1s})	SO_3 wt. %	S ppm	S SD (%)	S DL (ppm^{-1s})
LCO-1 p1_t2_C Line 004		2.74	7.07	432.13	0.08	88.82	288.36	<LOD	<LOD	100.00	77.01	0.10	423.33	3.05	8.22	0.02	96.11	9.01	7.94
LCO-1 p1_t2_C Line 005		3.04	6.41	335.24	<LOD	100.00	341.09	<LOD	<LOD	100.00	78.00	0.11	484.43	2.74	8.13	0.03	120.14	7.19	7.86
LCO-1 p1_t2_C Line 006		2.87	6.56	314.85	0.15	54.83	357.16	<LOD	<LOD	100.00	77.96	0.11	484.43	2.73	8.10	0.03	120.14	7.24	7.90
LCO-1 p1_t2_C Line 007	5	2.65	6.93	343.46	<LOD	100.00	347.98	<LOD	<LOD	100.00	77.65	0.16	689.55	2.13	8.15	0.03	132.15	6.65	7.76
LCO-1 p1_t2_C Line 008		2.67	6.93	353.50	<LOD	100.00	389.46	<LOD	<LOD	100.00	78.03	0.07	301.13	4.03	8.36	0.03	112.13	7.77	7.85
LCO-1 p1_t2_D Line 006		2.95	6.63	385.85	0.18	42.07	297.93	<LOD	<LOD	100.00	78.47	0.12	536.80	2.56	8.24	0.03	132.15	6.78	8.00
LCO-1 p1_t2_D Line 007		3.22	6.19	334.22	<LOD	100.00	325.57	<LOD	<LOD	100.00	78.66	0.14	624.08	2.29	8.18	0.04	160.18	5.63	7.86
**AVERAGE**		**2.88**			**0.14**			**<LOD**	**<LOD**			**0.12**	**506.25**			**0.03**	**124.71**		
**1σ SD**		**0.21**			**0.05**			**NA**	**NA**			**0.03**	**128.01**			**0.00**	**19.95**		
LCO-1 p1_t2_D Line 002	6	1.92	8.81	438.95	<LOD	100.00	399.28	<LOD	<LOD	100.00	78.84	0.09	405.87	3.14	8.25	0.03	108.12	8.15	7.98
**AVERAGE**		**1.92**			**<LOD**			**<LOD**	**<LOD**			**0.09**	**405.87**			**0.03**	**108.12**		
**1σ SD**		**NA**			**NA**			**NA**	**NA**			**NA**	**NA**			**NA**	**NA**		
LCO-1 p2_t3 E Line 004		5.92	4.50	394.34	0.27	36.11	384.45	<LOD	<LOD	100.00	94.83	0.20	872.84	1.85	8.82	0.02	72.08	12.17	8.42
LCO-1 p2_t3 E Line 005		6.52	4.22	335.24	0.23	40.52	382.19	<LOD	<LOD	100.00	85.66	0.22	942.67	1.72	8.25	0.05	212.24	4.40	8.02
LCO-1 p2_t3 E Line 006		7.70	3.90	386.25	0.09	97.27	402.53	<LOD	<LOD	100.00	91.15	0.48	2086.09	1.05	8.49	0.06	248.28	3.90	8.20
LCO-1 p2_t3 E Line 007	7	5.07	4.88	377.41	0.19	43.97	318.20	<LOD	<LOD	100.00	82.89	0.39	1697.68	1.19	8.45	0.05	220.25	4.27	8.04
LCO-1 p2_t3 F Line 005		5.74	4.52	333.20	<LOD	100.00	371.91	<LOD	<LOD	100.00	84.45	0.47	2059.91	1.06	8.26	0.09	360.41	2.86	8.17
LCO-1 p2_t3 F Line 006		5.45	4.68	368.94	0.26	38.53	399.28	<LOD	<LOD	100.00	96.66	0.60	2622.89	0.93	9.10	0.08	308.35	3.36	8.51
LCO-1 p2_t3 F Line 007		3.95	5.69	423.58	<LOD	100.00	335.37	<LOD	<LOD	100.00	88.96	0.54	2352.31	0.98	8.65	0.04	156.18	5.93	8.26
**AVERAGE**		**5.77**			**0.21**			**<LOD**	**<LOD**			**0.41**	**1804.91**			**0.06**	**225.40**		
**1σ SD**		**1.17**			**0.07**			**NA**	**NA**			**0.15**	**675.34**			**0.02**	**94.98**		
LCO-1 p1t4_G Line 008	8	2.63	7.09	385.85	0.25	33.75	304.36	<LOD	<LOD	100.00	84.45	0.03	122.20	8.65	8.55	0.01	24.03	33.78	7.92
LCO-1 p1t4_G Line 009		7.10	4.07	377.41	0.21	41.50	349.22	<LOD	<LOD	100.00	75.51	0.02	100.38	10.47	8.75	0.00	16.02	48.31	8.13
**AVERAGE**		**4.86**			**0.23**			**<LOD**	**<LOD**			**0.03**	**111.29**			**0.01**	**20.02**		
**1σ SD**		**3.16**			**0.03**			**NA**	**NA**			**0.00**	**15.43**			**0.00**	**5.66**		

Table S11. Cont.

Sample ID	melt inclusion ID	Cl wt. %	Cl ppm	Cl SD (%)	Cl DL (ppm^{-1s})	CuO wt. %	Cu ppm	Cu SD (%)	Cu DL (ppm^{-1s})	TiO_3 wt. %	Ti ppm	Ti SD (%)	Ti DL (ppm^{-1s})	Totals %
LCO-1 p1_t2_C Line 004		0.14	1450.00	1.53	6.20	<LOD	<LOD	100.00	23.13	0.77	4596.96	0.95	16.05	99.02
LCO-1 p1_t2_C Line 005		0.15	1500.00	1.51	6.32	0.02	143.80	30.60	22.99	0.79	4740.80	0.93	15.97	99.06
LCO-1 p1_t2_C Line 006		0.15	1470.00	1.51	6.07	0.01	87.88	52.15	23.16	0.77	4596.96	0.95	16.15	99.03
LCO-1 p1_t2_C Line 007	5	0.14	1450.00	1.53	6.21	<LOD	<LOD	100.00	22.98	0.75	4483.09	0.96	16.08	99.05
LCO-1 p1_t2_C Line 008		0.12	1230.00	1.74	6.38	0.01	47.93	85.18	23.14	0.49	2960.75	1.25	15.88	98.69
LCO-1 p1_t2_D Line 006		0.11	1080.00	1.88	6.20	0.02	167.76	25.72	22.91	0.52	3140.56	1.20	15.83	98.79
LCO-1 p1_t2_D Line 007		0.13	1330.00	1.62	6.18	0.01	103.85	43.20	23.24	0.90	5370.11	0.85	15.59	99.18
**AVERAGE**		**0.14**	**1358.57**			**0.01**	**110.24**			**0.71**	**4269.89**			
**1σ SD**		**0.02**	**154.97**			**0.01**	**47.06**			**0.15**	**883.26**			
LCO-1 p1_t2_D Line 002	6	0.08	840.00	2.26	6.29	0.02	159.77	26.92	22.94	0.59	3536.12	1.12	16.02	98.78
**AVERAGE**		**0.08**	**840.00**			**0.02**	**159.77**			**0.59**	**3536.12**			
**1σ SD**		**NA**	**NA**			**NA**	**NA**			**NA**	**NA**			
LCO-1 p2_t3 E Line 004		0.07	690.00	2.67	6.47	<LOD	<LOD	100.00	24.26	1.04	6209.19	0.79	16.94	98.37
LCO-1 p2_t3 E Line 005		0.13	1310.00	1.64	6.26	0.03	239.66	18.64	23.47	0.99	5933.49	0.81	16.06	98.55
LCO-1 p2_t3 E Line 006		0.18	1840.00	1.32	6.41	0.03	255.64	17.97	23.77	2.16	12933.82	0.52	16.46	100.01
LCO-1 p2_t3 E Line 007	7	0.21	2100.00	1.22	6.41	0.03	255.64	17.38	23.36	1.61	9661.41	0.61	16.50	99.44
LCO-1 p2_t3 F Line 005		0.18	1820.00	1.33	6.43	0.03	223.68	20.35	23.79	2.08	12484.31	0.53	16.47	100.00
LCO-1 p2_t3 F Line 006		0.15	1540.00	1.51	6.62	0.02	151.78	30.28	24.23	2.24	13413.29	0.51	16.91	100.14
LCO-1 p2_t3 F Line 007		0.15	1540.00	1.49	6.47	0.03	215.69	21.29	23.78	1.08	6484.89	0.77	16.49	98.94
**AVERAGE**		**0.15**	**1548.57**			**0.03**	**223.68**			**1.60**	**9588.63**			
**1σ SD**		**0.05**	**457.69**			**0.00**	**38.81**			**0.56**	**3381.87**			
LCO-1 p1t4_G Line 008	8	0.01	140.00	10.24	6.23	<LOD	<LOD	100.00	23.59	0.08	497.45	5.00	16.40	95.56
LCO-1 p1t4_G Line 009		0.01	60.00	23.26	6.30	0.01	79.89	53.57	23.33	0.08	449.51	5.36	16.03	95.61
**AVERAGE**		**0.01**	**100.00**			**0.01**	**79.89**			**0.08**	**473.48**			
**1σ SD**		**0.01**	**56.57**			**NA**	**NA**			**0.01**	**33.90**			

Table S11. Cont.

Sample ID	melt inclusion ID	K_2O wt. %	K SD (%)	K DL (ppm^{-1s})	CaO wt. %	Ca SD (%)	Ca DL (ppm^{-1s})	Na_2O wt. %	Na SD (%)	Na DL (ppm^{-1s})	Al_2O_3 wt. %	Al SD (%)	Al DL (ppm^{-1s})	SiO_2 wt. %	Si SD (%)	Si DL (ppm^{-1s})	MgO wt. %	Mg SD (%)	Mg DL (ppm^{-1s})
LCO-1 p1t5_I Line 004	9	4.86	2.22	104.81	2.48	3.23	124.53	3.79	5.72	261.93	12.26	2.10	344.65	67.67	0.87	378.41	0.83	10.01	193.86
LCO-1 p1t5_I Line 005		4.18	2.39	93.20	2.15	3.48	118.66	2.38	7.56	305.10	8.58	2.51	290.96	70.67	0.84	408.97	1.44	7.47	226.52
LCO-1 p1t5_I Line 006		4.84	2.25	122.32	1.13	5.05	121.81	2.79	6.65	221.41	10.34	2.26	288.49	74.51	0.82	424.43	0.82	10.55	246.58
LCO-1 p1t5_I Line 007		4.86	2.22	103.19	1.09	5.09	111.85	2.37	7.53	316.05	9.79	2.34	332.57	75.01	0.82	421.74	0.85	10.17	229.26
LCO-1 p1t5_J Line 003		4.26	2.37	96.16	2.56	3.16	121.74	1.70	8.77	212.02	9.19	2.43	308.08	69.27	0.85	412.86	1.82	6.62	249.04
**AVERAGE**		**4.60**			**1.88**			**2.60**			**10.03**			**71.42**			**1.15**		
**1σ SD**		**0.35**			**0.72**			**0.77**			**1.41**			**3.23**			**0.46**		
LCO-1 p1t6_K Line 004	10	4.66	2.27	106.40	1.23	4.66	100.55	3.15	6.27	230.56	10.05	2.30	286.42	71.48	0.84	397.06	0.86	9.98	209.75
LCO-1 p1t6_K Line 007		4.64	2.27	99.03	1.39	4.49	125.68	3.08	6.40	260.10	9.56	2.37	292.46	72.52	0.83	430.11	0.86	10.64	279.67
**AVERAGE**		**4.65**			**1.31**			**3.12**			**9.80**			**72.00**			**0.86**		
**1σ SD**		**0.01**			**0.12**			**0.05**			**0.35**			**0.74**			**0.01**		
LCO-1 p2t7_M Line 003	11	3.50	2.62	94.10	3.32	2.75	125.97	4.99	4.95	252.16	13.26	2.02	312.39	65.25	0.89	394.19	1.24	8.23	229.26
LCO-1 p2t7_N Line 002		2.83	2.90	80.28	4.43	2.33	103.82	3.91	5.64	263.62	16.37	1.81	351.44	62.69	0.91	400.15	0.83	10.42	232.52
LCO-1 p2t7_N Line 003		3.55	2.61	103.73	3.32	2.74	117.13	4.63	5.18	266.98	12.49	2.09	344.48	65.23	0.89	406.60	1.57	7.22	236.42
**AVERAGE**		**3.29**			**3.69**			**4.51**			**14.04**			**64.39**			**1.21**		
**1σ SD**		**0.40**			**0.64**			**0.55**			**2.06**			**1.47**			**0.37**		
LCO-1 p2t8_O Line 004	12	3.72	2.59	132.44	2.80	2.98	109.32	4.03	5.49	207.78	12.03	2.11	296.71	66.35	0.88	395.27	1.14	8.81	258.64
**AVERAGE**		**3.72**			**2.80**			**4.03**			**12.03**			**66.35**			**1.14**		
**1σ SD**		**NA**			**NA**			**NA**			**NA**			**NA**			**NA**		
LCO-1 p3t9_P Line 005	13	3.98	2.46	100.16	1.01	5.18	100.36	2.89	6.70	253.92	9.38	2.40	307.88	71.10	0.84	392.05	0.27	21.87	221.79
LCO-1 p3t9_P Line 006		4.42	2.36	125.27	1.42	4.36	109.89	3.33	6.09	260.10	10.59	2.23	304.59	74.22	0.82	451.61	0.17	33.77	250.84
LCO-1 p3t9_Q Line 004		2.28	3.30	102.64	4.09	2.45	120.62	6.60	4.29	380.67	19.37	1.65	375.01	62.66	0.92	408.86	<LOD	100.00	220.41
LCO-1 p3t9_Q Line 005		4.47	2.32	103.46	1.73	3.87	106.31	3.04	6.61	329.45	10.47	2.27	333.67	69.57	0.85	448.43	0.29	20.17	208.30
LCO-1 p3t9_Q Line 006		4.10	2.44	121.64	2.20	3.38	101.17	3.14	6.35	248.47	9.31	2.41	297.12	69.84	0.85	410.93	0.63	11.76	185.76
LCO-1 p3t9_Q Line 007		4.01	2.43	84.36	2.10	3.51	117.82	3.08	6.50	260.10	9.48	2.40	309.89	68.78	0.86	427.00	0.48	14.46	213.32
**AVERAGE**		**3.88**			**2.09**			**3.68**			**11.43**			**69.36**			**0.37**		
**1σ SD**		**0.81**			**1.07**			**1.44**			**3.93**			**3.80**			**0.19**		

Notes: any analyses that are less than the minimum detection limit (LOD) are listed as <LOD, and the respective errors for those analyses are expressed at 100%. SD – standard deviation; DL – detection limit

Table S11. Cont.

Sample ID	melt inclusion ID	FeO_{tot} wt. %	Fe SD (%)	Fe DL (ppm^{-1s})	MnO wt. %	Mn SD (%)	Mn DL (ppm^{-1s})	F wt. %	F ppm	F SD (%)	F DL (ppm^{-1s})	P_2O_5 wt. %	P ppm	P SD (%)	P DL (ppm^{-1s})	SO_3 wt. %	S ppm	S SD (%)	S DL (ppm^{-1s})
LCO-1 p1t5_I Line 004	9	3.37	6.09	360.36	0.17	46.85	314.82	<LOD	<LOD	100.00	75.76	0.05	235.67	4.80	8.22	0.01	44.05	19.02	7.91
LCO-1 p1t5_I Line 005		4.89	4.99	384.48	<LOD	100.00	355.95	<LOD	<LOD	100.00	80.15	0.28	1213.25	1.46	8.34	0.05	188.22	4.88	8.06
LCO-1 p1t5_I Line 006		2.76	6.77	334.96	<LOD	100.00	353.50	<LOD	<LOD	100.00	83.75	0.08	331.68	3.61	8.11	0.02	72.08	11.73	7.88
LCO-1 p1t5_I Line 007		2.94	6.58	360.79	0.15	50.39	300.83	<LOD	<LOD	100.00	78.36	0.07	288.04	4.10	8.22	0.04	148.17	5.90	7.74
LCO-1 p1t5_J Line 003		5.07	4.88	377.57	0.30	28.54	307.20	<LOD	<LOD	100.00	85.89	0.08	331.68	3.74	8.56	0.01	48.06	17.04	8.05
**AVERAGE**		**3.81**			**0.20**			**<LOD**	**<LOD**			**0.11**	**480.06**			**0.02**	**100.11**		
**1σ SD**		**1.10**			**0.09**			**NA**	**NA**			**0.09**	**411.76**			**0.02**	**64.63**		
LCO-1 p1t6_K Line 004	10	3.78	5.75	378.06	<LOD	100.00	318.20	<LOD	<LOD	100.00	77.08	0.12	514.98	2.64	8.13	0.04	168.19	5.41	7.92
LCO-1 p1t6_K Line 007		3.94	5.56	343.73	<LOD	100.00	360.14	<LOD	<LOD	100.00	66.95	0.09	370.96	3.10	6.97	0.01	44.05	15.04	6.40
**AVERAGE**		**3.86**			**<LOD**			**<LOD**	**<LOD**			**0.10**	**442.97**			**0.03**	**106.12**		
**1σ SD**		**0.12**						**NA**	**NA**			**0.02**	**101.84**			**0.02**	**87.78**		
LCO-1 p2t7_M Line 003	11	4.40	5.33	416.73	<LOD	100.00	389.46	<LOD	<LOD	100.00	74.43	0.03	139.65	7.57	8.40	0.01	56.06	14.69	7.97
LCO-1 p2t7_N Line 002		4.01	5.52	352.98	<LOD	100.00	366.67	<LOD	<LOD	100.00	76.28	0.05	235.67	4.89	8.39	0.02	96.11	9.07	7.98
LCO-1 p2t7_N Line 003		4.86	4.91	304.44	0.08	86.74	307.20	<LOD	<LOD	100.00	84.26	0.13	576.08	2.41	8.21	0.19	760.87	1.59	7.93
**AVERAGE**		**4.43**			**0.08**			**<LOD**	**<LOD**			**0.07**	**317.13**			**0.08**	**304.35**		
**1σ SD**		**0.43**			**NA**			**NA**	**NA**			**0.05**	**229.33**			**0.10**	**395.87**		
LCO-1 p2t8_O Line 004	12	4.17	5.47	401.97	<LOD	100.00	358.94	<LOD	<LOD	100.00	73.43	0.02	69.83	14.25	8.46	0.01	44.05	17.94	7.91
**AVERAGE**		**4.17**			**<LOD**			**<LOD**	**<LOD**			**0.02**	**69.83**			**0.01**	**44.05**		
**1σ SD**		**NA**			**NA**			**NA**	**NA**			**NA**	**NA**			**NA**	**NA**		
LCO-1 p3t9_P Line 005	13	6.74	4.20	393.01	<LOD	100.00	375.40	<LOD	<LOD	100.00	75.97	0.08	349.14	3.48	8.07	0.01	52.06	15.69	7.86
LCO-1 p3t9_P Line 006		1.94	8.66	416.95	<LOD	100.00	408.87	<LOD	<LOD	100.00	76.01	0.04	187.66	5.96	8.41	0.01	32.04	26.47	7.77
LCO-1 p3t9_Q Line 004		1.28	11.23	408.69	<LOD	100.00	345.47	<LOD	<LOD	100.00	86.70	0.21	907.76	1.78	8.40	0.01	32.04	27.63	8.32
LCO-1 p3t9_Q Line 005		4.13	5.55	431.12	0.17	50.36	357.72	<LOD	<LOD	100.00	77.94	0.09	384.05	3.25	8.16	0.00	20.02	40.04	8.02
LCO-1 p3t9_Q Line 006		4.61	5.06	314.85	0.19	41.91	322.27	<LOD	<LOD	100.00	87.13	0.32	1418.37	1.35	8.76	0.02	76.09	11.42	8.01
LCO-1 p3t9_Q Line 007		6.97	4.11	378.31	0.15	54.86	347.98	<LOD	<LOD	100.00	104.61	0.04	192.03	5.88	8.52	0.00	16.02	44.77	7.89
**AVERAGE**		**4.28**			**0.17**			**<LOD**	**<LOD**			**0.13**	**573.17**			**0.01**	**38.04**		
**1σ SD**		**2.36**			**0.02**			**NA**	**NA**			**0.11**	**491.15**			**0.01**	**22.48**		

Table S11. Cont.

Sample ID	melt inclusion ID	Cl wt. %	Cl ppm	Cl SD (%)	Cl DL (ppm^{-1s})	CuO wt. %	Cu ppm	Cu SD (%)	Cu DL (ppm^{-1s})	TiO_3 wt. %	Ti ppm	Ti SD (%)	Ti DL (ppm^{-1s})	Totals %
LCO-1 p1t5_I Line 004	9	0.04	440.00	3.69	6.16	<LOD	<LOD	100.00	23.00	0.30	1792.04	1.77	16.06	96.03
LCO-1 p1t5_I Line 005		0.09	910.00	2.11	6.15	0.02	175.75	25.06	23.46	1.22	7300.00	0.72	16.36	97.24
LCO-1 p1t5_I Line 006		0.05	460.00	3.62	6.32	0.02	159.77	27.19	23.08	0.54	3230.46	1.18	16.06	96.32
LCO-1 p1t5_I Line 007		0.05	450.00	3.62	6.19	0.01	103.85	42.41	23.01	0.41	2457.31	1.41	15.93	96.17
LCO-1 p1t5_J Line 003		0.06	560.00	3.03	6.04	<LOD	<LOD	100.00	23.18	0.37	2205.58	1.53	16.09	96.07
**AVERAGE**		**0.06**	**564.00**			**0.02**	**146.46**			**0.57**	**3397.08**			
**1σ SD**		**0.02**	**199.32**			**0.00**	**37.75**			**0.37**	**2243.85**			
LCO-1 p1t6_K Line 004	10	0.17	1690.00	1.38	6.12	0.01	47.93	95.91	23.36	0.76	4578.98	0.94	16.10	106.38
LCO-1 p1t6_K Line 007		0.05	490.00	2.73	3.97	0.01	103.85	38.65	21.25	0.71	4267.32	0.93	12.01	96.38
**AVERAGE**		**0.11**	**1090.00**			**0.01**	**75.89**			**0.74**	**4423.15**			
**1σ SD**		**0.08**	**848.53**			**0.00**	**39.54**			**0.04**	**220.38**			
LCO-1 p2t7_M Line 003	11	0.04	370.00	4.37	6.29	<LOD	<LOD	100.00	23.34	0.16	946.96	2.87	15.89	95.83
LCO-1 p2t7_N Line 002		0.05	530.00	3.21	6.34	<LOD	<LOD	100.00	23.39	0.24	1444.42	2.09	16.10	95.93
LCO-1 p2t7_N Line 003		0.20	1980.00	1.25	6.28	0.04	279.60	16.17	23.26	1.11	6646.71	0.76	16.02	97.14
**AVERAGE**		**0.10**	**960.00**			**0.04**	**279.60**			**0.50**	**3012.70**			
**1σ SD**		**0.09**	**886.96**			**NA**	**NA**			**0.53**	**3156.96**			
LCO-1 p2t8_O Line 004	12	0.01	80.00	16.45	6.24	<LOD	<LOD	100.00	23.20	0.03	173.81	13.40	16.55	94.26
**AVERAGE**		**0.01**	**80.00**			**<LOD**	**<LOD**			**0.03**	**173.81**			
**1σ SD**		**NA**	**NA**			**NA**	**NA**			**NA**	**NA**			
LCO-1 p3t9_P Line 005	13	0.07	680.00	2.63	6.14	<LOD	<LOD	100.00	22.93	1.06	6335.05	0.78	16.00	96.41
LCO-1 p3t9_P Line 006		0.01	150.00	9.14	6.04	<LOD	<LOD	100.00	23.01	0.27	1612.23	1.88	15.37	95.53
LCO-1 p3t9_Q Line 004		0.09	920.00	2.13	6.29	<LOD	<LOD	100.00	23.79	3.25	19484.64	0.42	16.30	98.68
LCO-1 p3t9_Q Line 005		0.08	750.00	2.44	6.13	0.01	95.86	46.32	22.69	1.09	6544.82	0.76	15.74	96.44
LCO-1 p3t9_Q Line 006		0.10	1040.00	1.96	6.36	0.03	207.70	21.91	23.53	2.24	13401.31	0.51	16.57	97.83
LCO-1 p3t9_Q Line 007		0.02	180.00	7.68	5.97	<LOD	<LOD	100.00	22.39	0.25	1474.38	2.00	15.30	95.41
**AVERAGE**		**0.06**	**620.00**			**0.02**	**151.78**			**1.36**	**8142.07**			
**1σ SD**		**0.04**	**374.54**			**0.01**	**79.08**			**1.18**	**7060.12**			

Table S11. Cont.

Sample ID	melt inclusion ID	K_2O wt. %	K SD (%)	K DL (ppm^{-1s})	CaO wt. %	Ca SD (%)	Ca DL (ppm^{-1s})	Na_2O wt. %	Na SD (%)	Na DL (ppm^{-1s})	Al_2O_3 wt. %	Al SD (%)	Al DL (ppm^{-1s})	SiO_2 wt. %	Si SD (%)	Si DL (ppm^{-1s})	MgO wt. %	Mg SD (%)	Mg DL (ppm^{-1s})
LCO-1 p1t10_R Line 003	14	4.35	2.34	95.87	1.98	3.60	109.32	3.43	5.99	248.47	8.83	2.46	287.85	73.99	0.82	446.46	0.67	11.71	221.79
**AVERAGE**		**4.35**			**1.98**			**3.43**			**8.83**			**73.99**			**0.67**		
**1σ SD**		**NA**			**NA**			**NA**			**NA**			**NA**			**NA**		
LCO-1 p1t10_S Line 002	15	3.21	2.76	109.51	4.14	2.43	114.16	5.34	4.78	314.64	15.41	1.86	348.69	65.31	0.89	404.67	0.39	16.44	206.83
LCO-1 p1t10_S Line 003		2.97	2.87	107.97	5.05	2.20	125.68	4.59	5.06	193.03	16.48	1.79	339.36	62.58	0.91	389.73	0.38	17.45	240.26
**AVERAGE**		**3.09**			**4.60**			**4.96**			**15.94**			**63.94**			**0.39**		
**1σ SD**		**0.17**			**0.65**			**0.53**			**0.75**			**1.93**			**0.00**		
LCO-9 1_A Line 003	16	1.99	3.61	115.73	1.67	3.96	112.73	2.78	6.74	298.36	18.59	1.67	337.60	74.38	0.83	429.60	0.82	9.83	184.98
LCO-9 1_B Line 003		2.25	3.30	98.18	6.04	2.00	125.39	5.50	4.68	276.23	19.23	1.66	349.69	61.33	0.93	398.72	0.69	11.35	206.83
**AVERAGE**		**2.12**			**3.86**			**4.14**			**18.91**			**67.85**			**0.76**		
**1σ SD**		**0.18**			**3.09**			**1.92**			**0.45**			**9.23**			**0.09**		
LCO-9 2_C Line 003	17	1.55	3.94	76.71	8.46	1.68	128.66	5.06	4.93	316.05	21.47	1.58	409.59	57.18	0.96	403.85	1.00	9.50	256.90
LCO-9 2_C Line 004		1.87	3.61	85.68	6.44	1.92	109.65	4.67	5.24	344.27	17.85	1.74	351.76	57.90	0.95	394.57	1.74	6.81	240.26
LCO-9 2_D Line 003		3.42	2.67	112.54	2.98	2.91	124.59	3.94	5.66	295.25	12.47	2.08	334.90	67.73	0.87	381.77	1.50	7.46	258.64
**AVERAGE**		**2.28**			**5.96**			**4.56**			**17.27**			**60.94**			**1.42**		
**1σ SD**		**1.00**			**2.77**			**0.57**			**4.53**			**5.90**			**0.38**		
LCO-9 3_E Line 002	18	1.72	3.79	90.76	7.35	1.80	117.75	4.97	4.90	225.54	19.82	1.64	344.30	58.88	0.95	416.74	1.65	7.09	269.09
LCO-9 3_E Line 003		2.96	2.86	101.82	4.58	2.30	110.39	4.36	5.27	214.11	14.15	1.95	327.45	63.67	0.90	414.75	2.34	5.76	247.17
LCO-9 3_E Line 004		3.25	2.73	107.19	3.41	2.67	97.54	4.36	5.31	278.63	13.90	1.96	329.82	68.36	0.87	371.48	1.64	7.15	279.10
LCO-9 3_F Line 002		3.40	2.67	102.10	2.59	3.10	104.42	4.60	5.13	252.16	12.90	2.04	319.99	70.60	0.85	410.15	1.41	7.66	251.47
LCO-9 3_F Line 003		3.28	2.70	87.94	3.83	2.54	119.27	4.88	4.98	260.10	14.78	1.90	319.61	65.25	0.89	406.60	1.57	7.17	238.38
LCO-9 3_F Line 004		2.65	3.02	98.75	5.19	2.15	114.48	4.40	5.33	271.23	13.51	2.02	362.08	59.89	0.93	375.82	2.22	5.89	214.75
**AVERAGE**		**2.88**			**4.49**			**4.60**			**14.84**			**64.44**			**1.81**		
**1σ SD**		**0.63**			**1.67**			**0.27**			**2.52**			**4.61**			**0.38**		

Notes: any analyses that are less than the minimum detection limit (LOD) are listed as <LOD, and the respective errors for those analyses are expressed at 100%; SD – standard deviation; DL – detection limit

Table S11. Cont.

Sample ID	melt inclusion ID	FeO$_{tot}$ wt. %	Fe SD (%)	Fe DL (ppm^{-1s})	MnO wt. %	Mn SD (%)	Mn DL (ppm^{-1s})	F wt. %	F ppm	F SD (%)	F DL (ppm^{-1s})	P$_2$O$_5$ wt. %	P ppm	P SD (%)	P DL (ppm^{-1s})	SO$_3$ wt. %	S ppm	S SD (%)	S DL (ppm^{-1s})
LCO-1 p1t10_R Line 003	14	2.70	6.82	324.92	<LOD	100.00	377.08	<LOD	<LOD	100.00	75.74	0.01	34.91	27.14	8.53	0.00	8.01	84.92	7.99
**AVERAGE**		**2.70**			**<LOD**			**<LOD**	**<LOD**			**0.01**	**34.91**			**0.00**	**8.01**		
**1σ SD**		**NA**			**NA**			**NA**	**NA**			**NA**	**NA**			**NA**	**NA**		
LCO-1 p1t10_S Line 002	15	1.87	8.48	343.19	<LOD	100.00	357.72	<LOD	<LOD	100.00	74.42	0.01	65.46	15.64	8.42	0.01	24.03	35.36	7.97
LCO-1 p1t10_S Line 003		1.74	8.63	293.88	<LOD	100.00	346.73	<LOD	<LOD	100.00	80.64	0.09	405.87	3.13	8.17	0.01	52.06	15.05	7.70
**AVERAGE**		**1.81**			**<LOD**			**<LOD**	**<LOD**			**0.05**	**235.67**			**0.01**	**38.04**		
**1σ SD**		**0.09**						**NA**	**NA**			**0.06**	**240.71**			**0.00**	**19.82**		
LCO-9 1_A Line 003	16	1.39	10.37	370.36	<LOD	100.00	339.82	<LOD	<LOD	100.00	74.38	0.02	78.56	12.88	8.34	<LOD	<LOD	100.00	8.13
LCO-9 1_B Line 003		2.45	7.47	409.53	<LOD	100.00	371.91	<LOD	<LOD	100.00	90.19	0.01	61.10	17.61	8.63	<LOD	<LOD	100.00	8.11
**AVERAGE**		**1.92**			**<LOD**			**<LOD**	**<LOD**			**0.02**	**69.83**			**<LOD**	**<LOD**		
**1σ SD**		**0.75**						**NA**	**NA**			**0.00**	**12.34**			**NA**	**NA**		
LCO-9 2_C Line 003		2.38	7.76	453.05	<LOD	100.00	342.36	<LOD	<LOD	100.00	74.45	0.04	161.48	6.79	8.45	0.00	16.02	50.95	8.01
LCO-9 2_C Line 004	17	4.96	4.92	369.44	<LOD	100.00	422.36	<LOD	<LOD	100.00	82.09	0.21	925.21	1.74	8.18	0.01	44.05	19.33	8.08
LCO-9 2_D Line 003		4.33	5.35	401.82	<LOD	100.00	367.85	<LOD	<LOD	100.00	80.92	0.09	410.24	3.13	8.45	0.01	32.04	27.49	8.12
**AVERAGE**		**3.89**			**<LOD**			**<LOD**	**<LOD**			**0.11**	**498.97**			**0.01**	**30.70**		
**1σ SD**		**1.35**						**NA**	**NA**			**0.09**	**389.52**			**0.00**	**14.06**		
LCO-9 3_E Line 002		2.85	6.66	352.27	<LOD	100.00	320.92	<LOD	<LOD	100.00	85.74	0.01	52.37	18.47	8.32	0.00	16.02	56.29	8.04
LCO-9 3_E Line 003		4.37	5.33	408.69	<LOD	100.00	380.52	<LOD	<LOD	100.00	85.97	0.31	1339.81	1.39	8.56	0.03	100.11	8.88	8.14
LCO-9 3_E Line 004	18	3.65	5.83	369.19	0.13	59.98	334.07	<LOD	<LOD	100.00	81.81	0.29	1269.99	1.42	8.27	0.00	16.02	50.68	8.05
LCO-9 3_F Line 002		3.04	6.53	394.11	<LOD	100.00	344.21	<LOD	<LOD	100.00	85.51	0.40	1745.68	1.17	8.45	0.02	64.07	12.93	8.00
LCO-9 3_F Line 003		3.12	6.48	409.75	<LOD	100.00	364.29	<LOD	<LOD	100.00	92.25	0.44	1942.07	1.10	8.57	<LOD	<LOD	100.00	8.13
LCO-9 3_F Line 004		5.30	4.64	249.28	<LOD	100.00	300.83	<LOD	<LOD	100.00	72.55	0.02	82.92	12.49	8.52	0.00	20.02	41.39	7.98
**AVERAGE**		**3.72**			**0.13**			**<LOD**	**<LOD**			**0.25**	**1072.14**			**0.01**	**43.25**		
**1σ SD**		**0.95**			**NA**			**NA**	**NA**			**0.19**	**817.28**			**0.01**	**37.72**		

Notes: any analyses that are less than the minimum detection limit (LOD) are listed as <LOD, and the respective errors for those analyses are expressed at 100%. SD – standard deviation; DL – detection limit

Table S11. Cont.

Sample ID	melt inclusion ID	Cl wt. %	Cl ppm	Cl SD (%)	Cl DL (ppm^{-1s})	CuO wt. %	Cu ppm	Cu SD (%)	Cu DL (ppm^{-1s})	TiO_3 wt. %	Ti ppm	Ti SD (%)	Ti DL (ppm^{-1s})	Totals %
LCO-1 p1t10_R Line 003	14	0.00	20.00	68.77	6.20	<LOD	<LOD	100.00	23.27	0.03	191.79	11.76	15.77	95.92
**AVERAGE**		**0.00**	**20.00**			**<LOD**	**<LOD**			**0.03**	**191.79**			
**1σ SD**		**NA**	**NA**			**NA**	**NA**			**NA**	**NA**			
LCO-1 p1t10_S Line 002	15	0.00	50.00	29.30	6.26	<LOD	<LOD	100.00	23.26	0.04	209.77	10.76	15.88	97.83
LCO-1 p1t10_S Line 003		0.11	1110.00	1.86	6.33	0.02	127.82	35.07	23.01	0.55	3302.38	1.15	15.43	98.53
**AVERAGE**		**0.06**	**580.00**			**0.02**	**127.82**			**0.29**	**1756.07**			
**1σ SD**		**0.07**	**749.53**			**NA**	**NA**			**0.36**	**2186.81**			
LCO-9 1_A Line 003	16	0.00	20.00	71.34	6.30	<LOD	<LOD	100.00	23.46	0.04	227.75	9.99	16.06	99.55
LCO-9 1_B Line 003		0.00	30.00	52.32	6.30	<LOD	<LOD	100.00	23.44	0.05	269.70	8.61	15.91	99.41
**AVERAGE**		**0.00**	**25.00**			**<LOD**	**<LOD**			**0.04**	**248.73**			
**1σ SD**		**0.00**	**7.07**			**NA**	**NA**			**0.00**	**29.67**			
LCO-9 2_C Line 003	17	0.00	10.00	95.77	6.32	<LOD	<LOD	100.00	23.26	0.07	443.51	5.54	16.36	96.38
LCO-9 2_C Line 004		0.00	50.00	27.37	6.30	<LOD	<LOD	100.00	23.67	0.48	2858.87	1.27	15.62	96.92
LCO-9 2_D Line 003		0.01	140.00	10.38	6.26	<LOD	<LOD	100.00	23.25	0.46	2756.98	1.31	15.75	96.79
**AVERAGE**		**0.01**	**66.67**			**<LOD**	**<LOD**			**0.34**	**2019.79**			
**1σ SD**		**0.01**	**66.58**			**NA**	**NA**			**0.23**	**1366.04**			
LCO-9 3_E Line 002	18	0.00	30.00	43.86	6.27	<LOD	<LOD	100.00	23.32	0.03	191.79	11.94	16.01	96.79
LCO-9 3_E Line 003		0.00	50.00	28.53	6.26	0.02	135.81	33.06	23.50	0.61	3661.99	1.10	16.30	97.74
LCO-9 3_E Line 004		0.01	80.00	18.11	6.24	0.03	207.70	21.49	23.28	0.99	5945.48	0.81	16.10	98.11
LCO-9 3_F Line 002		0.00	30.00	47.32	6.52	0.02	143.80	31.19	23.67	0.90	5394.09	0.86	16.37	98.11
LCO-9 3_F Line 003		0.00	40.00	35.86	6.34	0.03	199.72	22.50	23.56	0.71	4255.33	0.99	16.19	97.95
LCO-9 3_F Line 004		0.00	20.00	84.07	6.26	<LOD	<LOD	100.00	23.50	0.04	221.76	10.31	16.03	96.92
**AVERAGE**		**0.00**	**41.67**			**0.02**	**171.76**			**0.55**	**3278.41**			
**1σ SD**		**0.00**	**21.37**			**0.00**	**37.19**			**0.42**	**2512.42**			

Notes: any analyses that are less than the minimum detection limit (LOD) are listed as <LOD, and the respective errors for those analyses are expressed at 100%; SD – standard deviation; DL – detection limit

Table S11. Cont.

Sample ID	melt inclusion ID	K_2O wt. %	K SD (%)	K DL (ppm^{-1s})	CaO wt. %	Ca SD (%)	Ca DL (ppm^{-1s})	Na_2O wt. %	Na SD (%)	Na DL (ppm^{-1s})	Al_2O_3 wt. %	Al SD (%)	Al DL (ppm^{-1s})	SiO_2 wt. %	Si SD (%)	Si DL (ppm^{-1s})	MgO wt. %	Mg SD (%)	Mg DL (ppm^{-1s})
LCO-9 4_G Line 002	19	2.86	2.91	103.19	5.51	2.08	113.77	4.42	5.33	285.17	13.34	2.04	363.38	60.75	0.93	391.74	3.18	4.94	272.47
LCO-9 4_G Line 003		2.77	2.95	96.45	5.30	2.13	121.22	4.84	5.04	234.38	13.64	2.00	317.36	61.04	0.92	391.35	3.08	4.98	240.92
LCO-9 4_G Line 004		2.44	3.16	103.46	4.92	2.21	117.13	4.61	5.37	299.85	12.35	2.14	329.82	57.81	0.95	366.69	3.14	5.06	266.22
LCO-9 4_H Line 002		2.94	2.89	112.29	5.04	2.18	108.98	4.83	5.01	225.54	14.19	1.96	351.44	62.97	0.91	389.64	2.93	5.15	269.63
LCO-9 4_H Line 003		2.86	2.90	97.61	5.31	2.14	129.63	4.20	5.42	246.68	13.33	2.03	335.26	61.73	0.92	385.09	3.07	4.89	193.04
LCO-9 4_H Line 004		2.68	2.99	94.69	5.75	2.05	126.75	4.42	5.29	250.39	13.30	2.03	327.45	58.69	0.94	402.99	3.37	4.78	270.76
**AVERAGE**		**2.76**			**5.31**			**4.55**			**13.36**			**60.50**			**3.13**		
**1σ SD**		**0.18**			**0.30**			**0.25**			**0.60**			**1.92**			**0.14**		

Notes: any analyses that are less than the minimum detection limit (LOD) are listed as <LOD, and the respective errors for those analyses are expressed at 100%; SD – standard deviation; DL – detection limit

Table S11. Cont.

Sample ID	melt inclusion ID	FeO_{tot} wt. %	Fe SD (%)	Fe DL (ppm^{-1s})	MnO wt. %	Mn SD (%)	Mn DL (ppm^{-1s})	F wt. %	F ppm	F SD (%)	F DL (ppm^{-1s})	P_2O_5 wt. %	P ppm	P SD (%)	P DL (ppm^{-1s})	SO_3 wt. %	S ppm	S SD (%)	S DL (ppm^{-1s})
LCO-9 4_G Line 002		5.12	4.91	423.95	0.13	65.99	365.48	<LOD	<LOD	100.00	95.91	0.89	3866.69	0.74	9.16	<LOD	<LOD	100.00	8.38
LCO-9 4_G Line 003		5.77	4.57	402.43	0.09	90.94	360.14	<LOD	<LOD	100.00	89.96	0.91	3967.07	0.73	8.61	0.01	32.04	26.66	8.08
LCO-9 4_G Line 004	19	11.53	3.12	333.39	<LOD	100.00	369.03	<LOD	<LOD	100.00	90.19	0.97	4246.38	0.70	8.74	<LOD	<LOD	100.00	8.20
LCO-9 4_H Line 002		4.76	5.06	393.64	<LOD	100.00	349.22	0.02	240.00	55.53	86.64	1.01	4399.12	0.69	8.66	0.00	12.01	81.81	8.30
LCO-9 4_H Line 003		4.71	5.03	342.46	0.09	90.03	358.94	<LOD	<LOD	100.00	89.76	0.96	4211.46	0.70	8.66	0.00	12.01	65.82	8.23
LCO-9 4_H Line 004		5.02	4.84	325.20	0.17	48.91	334.07	<LOD	<LOD	100.00	90.35	0.80	3495.73	0.78	8.43	<LOD	<LOD	100.00	8.18
**AVERAGE**		**6.15**			**0.12**			**0.02**	**240.00**			**0.92**	**4031.07**			**0.00**	**18.69**		
**1σ SD**		**2.66**			**0.04**			**NA**	**NA**			**0.07**	**326.21**			**0.00**	**11.56**		

Notes: any analyses that are less than the minimum detection limit (LOD) are listed as <LOD, and the respective errors for those analyses are expressed at 100%; SD – standard deviation; DL – detection limit

Table S11. Cont.

Sample ID	melt inclusion ID	Cl wt. %	Cl ppm	Cl SD (%)	Cl DL (ppm^{-1s})	CuO wt. %	Cu ppm	Cu SD (%)	Cu DL (ppm^{-1s})	TiO_3 wt. %	Ti ppm	Ti SD (%)	Ti DL (ppm^{-1s})	Totals %
LCO-9 4_G Line 002	19	<LOD	<LOD	100.00	6.52	0.01	63.91	75.28	24.24	1.17	7018.31	0.73	16.18	97.76
LCO-9 4_G Line 003		0.00	20.00	81.48	6.36	0.02	191.73	23.06	23.51	1.01	6035.38	0.80	16.51	97.66
LCO-9 4_G Line 004		<LOD	<LOD	100.00	6.52	0.03	223.68	20.40	23.95	1.13	6748.60	0.75	16.54	97.83
LCO-9 4_H Line 002		<LOD	<LOD	100.00	6.58	0.04	311.56	14.61	23.84	1.15	6898.44	0.74	16.38	98.10
LCO-9 4_H Line 003		0.00	30.00	50.19	6.41	0.03	215.69	21.03	24.01	1.10	6604.76	0.76	16.60	97.81
LCO-9 4_H Line 004		0.00	40.00	30.50	6.15	0.02	175.75	25.86	23.65	0.81	4848.68	0.91	16.19	97.37
**AVERAGE**		**0.00**	**30.00**			**0.02**	**197.05**			**1.06**	**6359.03**			
**1σ SD**		**0.00**	**10.00**			**0.01**	**80.47**			**0.14**	**815.31**			

Notes: any analyses that are less than the minimum detection limit (LOD) are listed as <LOD, and the respective errors for those analyses are expressed at 100%; SD – standard deviation; DL – detection limit

Table S12. Whole-rock major and trace elements geochemistry of andesite LCO-9 with plagioclase phenocrysts hosting immiscible melt inclusions used in Figure 6c and Supplementary Figure S9C.

Sample		LCO-9	Methodology[1]
SiO_2	%	57.30	ME-ICP06
Al_2O_3	%	16.40	ME-ICP06
Fe_2O_3	%	7.12	ME-ICP06
CaO	%	6.63	ME-ICP06
MgO	%	3.96	ME-ICP06
Na_2O	%	3.46	ME-ICP06
K_2O	%	1.83	ME-ICP06
Cr_2O_3	%	0.01	ME-ICP06
TiO_2	%	0.89	ME-ICP06
MnO	%	0.11	ME-ICP06
P_2O_5	%	0.24	ME-ICP06
SrO	%	0.06	ME-ICP06
BaO	%	0.05	ME-ICP06
LOI	%	1.09	OA-GRA05
Total	%	99.15	
C	%	0.11	C-IR07
S	%	0.01	S-IR08
Ba	ppm	439.00	ME-MS81
Ce	ppm	53.60	ME-MS81
Cr	ppm	50.00	ME-MS81
Cs	ppm	1.32	ME-MS81
Dy	ppm	3.48	ME-MS81
Er	ppm	2.04	ME-MS81
Eu	ppm	1.31	ME-MS81
Ga	ppm	20.30	ME-MS81
Gd	ppm	4.11	ME-MS81
Ge	ppm	<5	ME-MS81
Hf	ppm	4.20	ME-MS81
Ho	ppm	0.70	ME-MS81
La	ppm	25.70	ME-MS81
Lu	ppm	0.27	ME-MS81
Nb	ppm	9.70	ME-MS81
Nd	ppm	25.10	ME-MS81
Pr	ppm	6.44	ME-MS81
Rb	ppm	57.60	ME-MS81
Sm	ppm	4.98	ME-MS81
Sn	ppm	2.00	ME-MS81
Sr	ppm	593.00	ME-MS81
Ta	ppm	0.60	ME-MS81
Tb	ppm	0.60	ME-MS81
Th	ppm	7.42	ME-MS81
Tm	ppm	0.31	ME-MS81
U	ppm	1.61	ME-MS81
V	ppm	170.00	ME-MS81
W	ppm	187.00	ME-MS81
Y	ppm	20.10	ME-MS81

Yb	ppm	1.85	ME-MS81
Zr	ppm	157.00	ME-MS81
Pb	ppm	11.00	ME-MS81
As	ppm	0.60	ME-MS42
Bi	ppm	0.03	ME-MS42
Hg	ppm	0.60	ME-MS42
In	ppm	0.02	ME-MS42
Re	ppm	0.00	ME-MS42
Sb	ppm	0.07	ME-MS42
Se	ppm	0.20	ME-MS42
Te	ppm	<0.01	ME-MS42
Tl	ppm	0.02	ME-MS42
Ag	ppm	<0.5	ME-4ACD81
Cd	ppm	0.50	ME-4ACD81
Co	ppm	42.00	ME-4ACD81
Cu	ppm	50.00	ME-4ACD81
Li	ppm	10.00	ME-4ACD81
Mo	ppm	1.00	ME-4ACD81
Ni	ppm	33.00	ME-4ACD81
Sc	ppm	17.00	ME-4ACD81
Zn	ppm	91.00	ME-4ACD81

[1] Method name according to ALS laboratory explained below.

Notes: Major and minor elements were determined using fused bead, followed by acid digestion, and then inductively-coupled plasma–atomic emission spectrometry (ICP-AES; ALS laboratory method ME-ICP06). Loss on ignition (LOI) was determined by heating in an induction furnace (ALS laboratory method OA-GRA05). Total carbon and total sulfur (wt. %) were analyzed by heating in an induction furnace (ALS laboratory method C-IR07 and S-IR08, respectively). A lithium borate flux fusion, followed by acid digestion, was done prior to analysis by inductively coupled plasma mass spectrometry (ICP-MS) for most of the trace elements (ALS laboratory method ME-MS81). As, Bi, Hg, In, Re, Sb, Se, Te, and Tl were separately determined after aqua regia digestion by ICP-MS (ALS laboratory method ME-MS42). Ag, Cd, Co, Cu, Li, Mo, Ni, Sc, Zn were digested in four acids and then analyzed by ICP-AES to (ALS laboratory method ME-4ACD81).

References:

1. De Angelis, S. M. H., Neill, O. K., Owen K. MINERAL: A program for the propagation of analytical uncertainty through mineral formula recalculations. *Comp. Geoscien.* **48**, 134-142 (2012).
2. Giaramita, M. J., Day, H. W. Error propagation in calculations of structural formulas. *Amer. Mineral.* **75**, 170-182 (1990).
3. Kamenetsky, V. S. *et al.* Magma chamber-scale liquid immiscibility in the siberian traps represented by melt pools in native iron. *Geology* **41**, 1091–1094 (2013).
4. Hou, T. *et al.* Immiscible hydrous Fe-Ca-P melt and the origin of iron oxide-apatite ore deposits. *Nat. Commun.* **9**, 1–8 (2018).
5. Tornos, F., Velasco, F. & Hanchar, J. M. The magmatic to magmatic-hydrothermal evolution of the El Laco deposit (Chile) and its implications for the genesis of magnetite-apatite deposits. *Econ. Geol.* **112**, 1595–1628 (2017).
6. Pietruszka, D. K., Hanchar, J. M., Tornos, F., Whitehouse, M. J., & Velasco, F. Tracking isotopic sources of immiscible melts at the enigmatic magnetite-(apatite) deposit at El Laco, Chile, using Pb isotopes. *Geol. Soc. Am. Bull*. 1–18 (2023). https://doi.org/10.1130/B36506.1
